# Joint downlink user association and interference avoidance with a load balancing approach in backhaul-constrained HetNets

**DOI:** 10.1371/journal.pone.0298352

**Published:** 2024-03-04

**Authors:** Maryam Chinipardaz, Somaieh Amraee, Ahmad Sarlak

**Affiliations:** 1 Department of Electrical and Computer Engineering, Jundi-Shapur University of Technology, Dezful, Iran; 2 Department of Electrical and Computer Engineering, K. N. Toosi University of Technology, Tehran, Iran; Khon Kaen University, THAILAND

## Abstract

In heterogeneous networks (HetNets), different lower-power base stations are added in a typically unplanned manner to the well-planned macro-only network, bringing new challenges to the network functions. Small cells experience limited backhaul capacity since cost-effective backhaul is not easily accessible to them. This study focuses on the issue of user association in backhaul-constrained HetNets. It shows that it is necessary to associate users with cells using a load balancing approach in order to fully leverage the addition of small cells. The cell association needs to be done jointly with an interference management technique that protects offloaded users and those prone to harmful interference. After modeling the system and describing the interference model, the problem of cell and subband allocation is formulated. We first examine the problem in a time-sharing mode and present a centralized heuristic solution to the cell and subband allocation problem. This is accomplished by solving the convex problem using the gradual removal method. The importance of providing distributed algorithms for HetNets leads to the development of a new algorithm through the application of the dual decomposition method to a reformulated problem and the use of an admission control mechanism. In the achieved algorithm, all computations are performed locally, with each user and base station relying only on local information. This algorithm obtains near-optimal answers, as confirmed by the simulation results. Compared with conventional cell allocation methods, our distributed algorithm prevents intensive interference for all users and achieves better load balance between network tiers, resulting in improved network utility.

## 1. Introduction

Due to the growing demands of cellular network users, there is a need to increase the capacity of these networks [1]. Developing a heterogeneous cellular network by increasing the spectral efficiency per unit area is a definite solution to increase the capacity of current and future generations of cellular networks [2, 3].

A multi-tier heterogeneous network (HetNet) is a network in which traditional well-planned macro base station (BS) deployment is overlaid by small-scale unplanned BSs, some of which may also have access restrictions or lack a wired backhaul network. Small-scale BSs in a heterogeneous LTE-A network are categorized as pico, femto, and relay stations, and their deployment is increasing significantly [4].

These networks have many advantages over macro-only cellular networks. In general, they can improve network capacity and coverage while reducing power consumption and cost [5, 6]. In this study, the BS deployment is of the co-channel type, in which all cells use the same frequency band. This method is commonly used because it involves a more cost-effective and efficient use of the spectrum [7]. However, due to bandwidth sharing among cells, the resulting interference needs to be dealt with using efficient interference management techniques [8].

In this study, orthogonal frequency division multiple access (OFDMA) is considered as the multiple access scheme, which is the dominant multiple access technology in 4G/5G networks in which users in a cell transmit on different subbands. This method prevents intra-cell and inter-symbol interference [9].

In multicellular networks, a user may be within the coverage area of multiple BSs, meaning that there are different BS connection options. The user association is, in fact, the method of selecting a suitable BS for the user according to the given parameters [10]. Cells can be selected using simple decision metrics, such as reference signal received power (RSRP), reference signal received quality (RSRQ), and signal to interference plus noise ratio (SINR) [7]. Research has shown that using these simple metrics to allocate cells can maximize network throughput in macro-only cellular networks with uniform traffic [11]. However, if the same method is used in HetNets, most users are connected to the macro BSs; therefore, the small-scale BSs would remain unused. This problem is called load imbalance and is one of the main causes of network performance degradation [12] as it disables the use of small cells in HetNets [5].

Therefore, it is necessary to apply load balancing methods in these networks to push users to connect to smaller BSs. These users, called offloaded users, are connected to smaller BSs despite receiving a stronger signal from the macro BS. As a result, they are exposed to intense interference. In addition, interference in HetNets is more intense than that in single-tier networks, and interference mitigation techniques face new challenges [13]. These factors demonstrate the need of these networks for efficient interference management methods.

**Fig 1:**
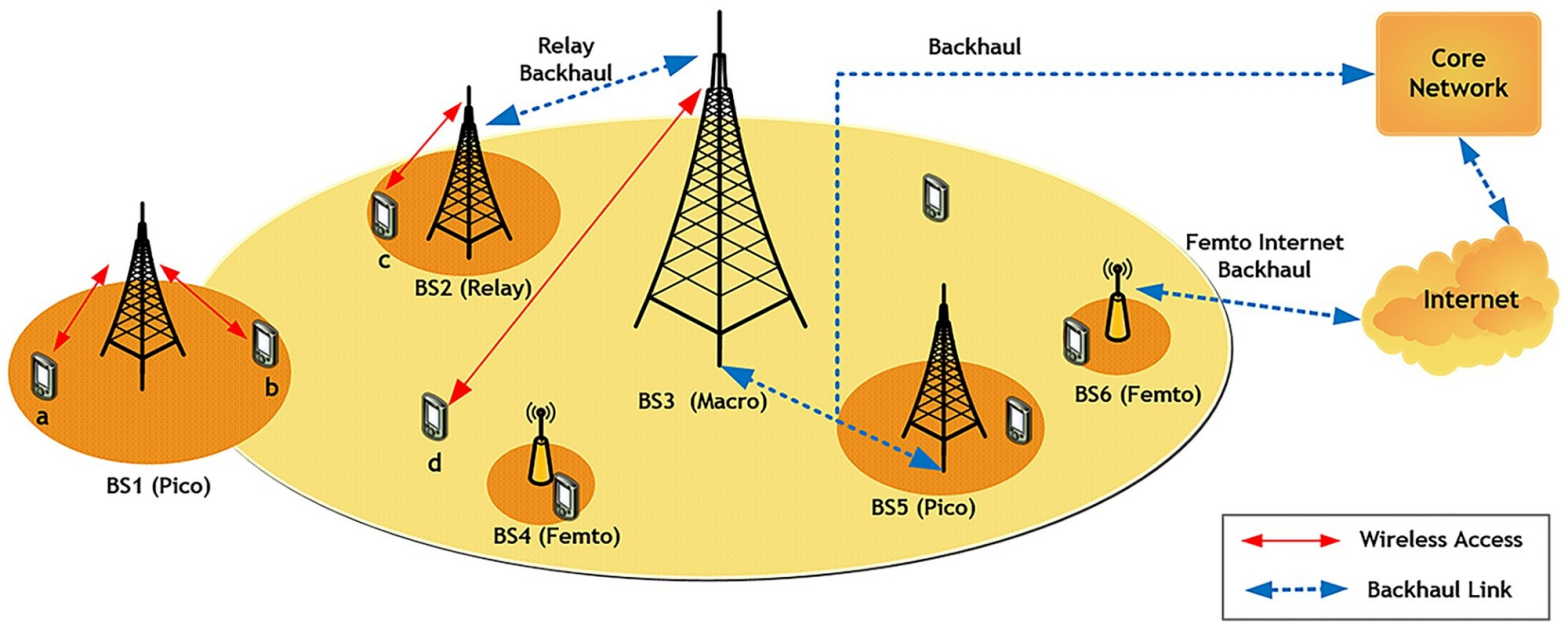
Illustration of a four-tier HetNet with different backhaul links.

The availability of ideal backhaul links with unlimited capacity is a common assumption in the current work on cell association, but this assumption is false for HetNets. Small-scale BSs could be set up by users indoors or in regions without backhaul network infrastructure [13]. Therefore, different backhaul solutions are adapted for smaller BSs (e.g., pico and femto), such as xDSL and non-line-of-sight microwaves, as opposed to ideal backhauls such as line-of-sight microwaves and optical fibers, due to economic benefits [14]. In this paper, the limited capacity for backhaul links of BSs is considered for load balancing in order to better model the difference between backhaul communication links of BSs in heterogeneous networks. In Fig 1, a four-tier HetNet is shown.

Although extensive studies on cell assignment and interference management in cellular networks have been conducted, the joint study of these two issues in backhaul-constrained HetNets is a currently developing topic. In this study, this issue is examined in detail, and effective solutions are proposed. The key contributions of this study are summarized as follows:

- The problem of allocating cells and subbands to users in backhaul-constrained HetNets is formulated, which jointly manages interference and balances the load among tiers in both the exclusive and time-sharing modes. In addition, the feasibility of a connection set is defined in both cases. In this model, by using a protocol interference model and a proper interference constraint, all network connections are protected against intensive interference.
- By solving the problem in time-sharing mode, a centralized algorithm for assigning cells and subbands in exclusive mode HetNets is proposed.
- After the problem is reformulated, an efficient iterative algorithm based on dual decomposition for assigning cells and subbands in multi-tier networks with a near-optimal response is proposed. In the proposed protocol, all BSs and users make use of local information and perform the required computations locally, which makes the method suitable for distributed implementation.
- A heuristic algorithm is presented for distributed admission control performed when a BS receives multiple requests for the same resource. This algorithm also helps balance the load among cells in the case of a high-connection request for a BS.

The remainder of this paper is organized as follows. In Section 2, load balancing and interference management issues in heterogeneous networks are described, and related works are reviewed. Then, the shortcomings of existing works that need to be addressed are highlighted. In Section 3, the network model and interference model are presented, and the problem of cell and subband association in heterogeneous networks is formulated. Section 4 includes the problem reformulations and the proposed algorithms. The simulation results used to evaluate the performance of the presented algorithms are presented in Section 5. Finally, in Section 6, some concluding remarks are provided.

## 2. Background and related works

The issues of load balancing and interference management, along with an overview of related works, are described in this section.

### 2.1. Load balancing

Load balancing has been considered in a wide variety of contexts [15]. Load balancing is an approach for resource allocation, according to which load is balanced among resources to optimize quantities, such as resource utilization, waiting/processing delays, fairness, or throughput [16]. In cellular networks, load balancing refers to the distribution of loads among cells in proportion to their resources [13]. For instance, the lowest traffic delivery latency or the highest green energy usage in networks with hybrid energy BSs are taken into account for load balancing functions in some studies [14, 17, 18]. In the case of load imbalance in multi-tier networks, the deployment of small-scale BSs is not beneficial, and the network performance is significantly reduced. Therefore, load balancing methods must be applied in HetNets.

There are two general strategies for balancing load in cellular networks [19]. One approach involves borrowing resources (such as frequency channels) from lightly-loaded cells to heavily-loaded cells, while the other approach focuses on transferring traffic from heavily-loaded cells to lightly-loaded cells. The latter strategy is appropriate and practical for co-channel deployment since all the cells have the same frequency subbands.

The proposed methods based on this strategy are divided into two general categories in the literature [13]. The first group includes heuristic methods that define a tier-based or cell-based criterion and claim that if the cell allocation in a network follows this criterion, load balancing occurs among cells. This load-based criterion can be provided to users by BSs. This is similar to the method of cell breathing, in which each BS increases or decreases the amount of its reference signal according to its load status [20, 21]. The user can provide a load-based criterion, such as the cell range expansion (CRE) method, where each user multiplies the amount of received power of a BS by its tier bias before making a decision [22].

The second group of methods for load balancing involves formulating the resource allocation problem as an optimization problem in such a way that load balancing occurs in response to the problem. Proper definition of the objective function can lead to load balancing. In the literature, for this purpose, the minimization of maximum cell load [10] and the minimization of cell load variance [23] have been used as objective functions. The work in [24] defines the concept of entropy for the network in such a way that the network load is distributed among the BSs by maximizing the entropy function.

Some other studies have used the network utility function in their formulated problems. However, its proper use in the objective function has a great deal to achieve in load balancing. It can be said that load balancing leads to the fair use of network resources by users. Accordingly, to balance the load, the max-min fairness is used in [25] to solve the problem of maximizing the minimum utility functions of users. Meanwhile, in [12, 19, 26], proportional fairness with maximizing the logarithmic sum of the users’ utility function has been used.

In general, the formulated problem of cell association falls within the scope of integer or mixed-integer programming, since the assignment decisions are typically modeled using binary or integer variables. Solving integer programming problems is known to be NP-hard, requiring more advanced algorithms or heuristics [2, 16]. Since the goal of this research is to find near-optimal solutions, the formulation method is used.

### 2.2. Interference management

In a network using OFDMA technology, the frequency subbands assigned to cell users are orthogonal; therefore, there is no intra-cell interference. Inter-cell interference can occur between cells of the same tier or cells of different tiers [6].

Interference is the most important source of network capacity reduction and achievable data rate limitation in wireless cellular networks [12, 27]. Therefore, inter-cell interference (ICI) management is one of the most critical challenges associated with multi-tier networks using OFDMA technology. HetNets encounter new challenges in managing interference compared with to single-tier networks, some of which are as follows:

- Load balancing is a necessity in HetNets. New scenarios of interference in the uplink and downlink are caused by load balancing, which increases the need for interference management, especially for offloaded users [8, 26].
- In multi-tier networks, some small-scale BSs have restrictive access policies that may create coverage holes in the network, thus increasing the complexity of interference management methods.
- Different tiers within HetNets use different backhaul networks that differ in terms of bandwidth and delays, and such differences should be considered in the interference management method.
- While only cell-edge users of macro cells are prone to interference in single-tier networks, users anywhere in the cell are prone to interference in HetNets. Therefore, HetNets require more comprehensive interference management.

Due to the new design of the physical layer in new-generation networks and OFDMA systems, the design of interference management techniques has become more flexible [6, 8]. Interference management methods are strategically divided into two general categories: interference avoidance techniques and interference diminishing techniques [13].

In the former, resources are allocated between the cells in a way that prevents intensive inter-cell interference; these resources include time, frequency, space, and power [28]. In order to prevent interference, assigning resources must be done by time partitioning, frequency partitioning, space partitioning, or controlling the transmission power. For example, Li et al. [29] divided the received signal space into the desired subspace and the interference subspaces, whereas frequency division was performed in [30]. Kurda [31] suggested the power control of femto cells in order to mitigate inter-cell interference in LTE-Advanced HetNets. In some studies, a combination of these methods has been used [32]. In the latter, the interference diminishing method requires coding techniques and special equipment on the BSs and the user side. Methods in this category can be divided into interference cancelation and interference randomization methods. Coding techniques are designed to diminish interference instead of avoiding it [6, 9]. These methods can be used in conjunction with resource allocation methods to improve interference management [8].

In this study, we use interference avoidance methods, which are also known as inter-cell resource allocation methods. Interference management methods using inter-cell resource allocation can be implemented centrally or distributed. In heterogeneous networks, providing distributed solutions for resource allocation is essential due to the unplanned and ad hoc deployment of small-scale BSs and differences in the backhaul connections of BSs [6, 8].

### 2.3. Related works

**Fig 2:**
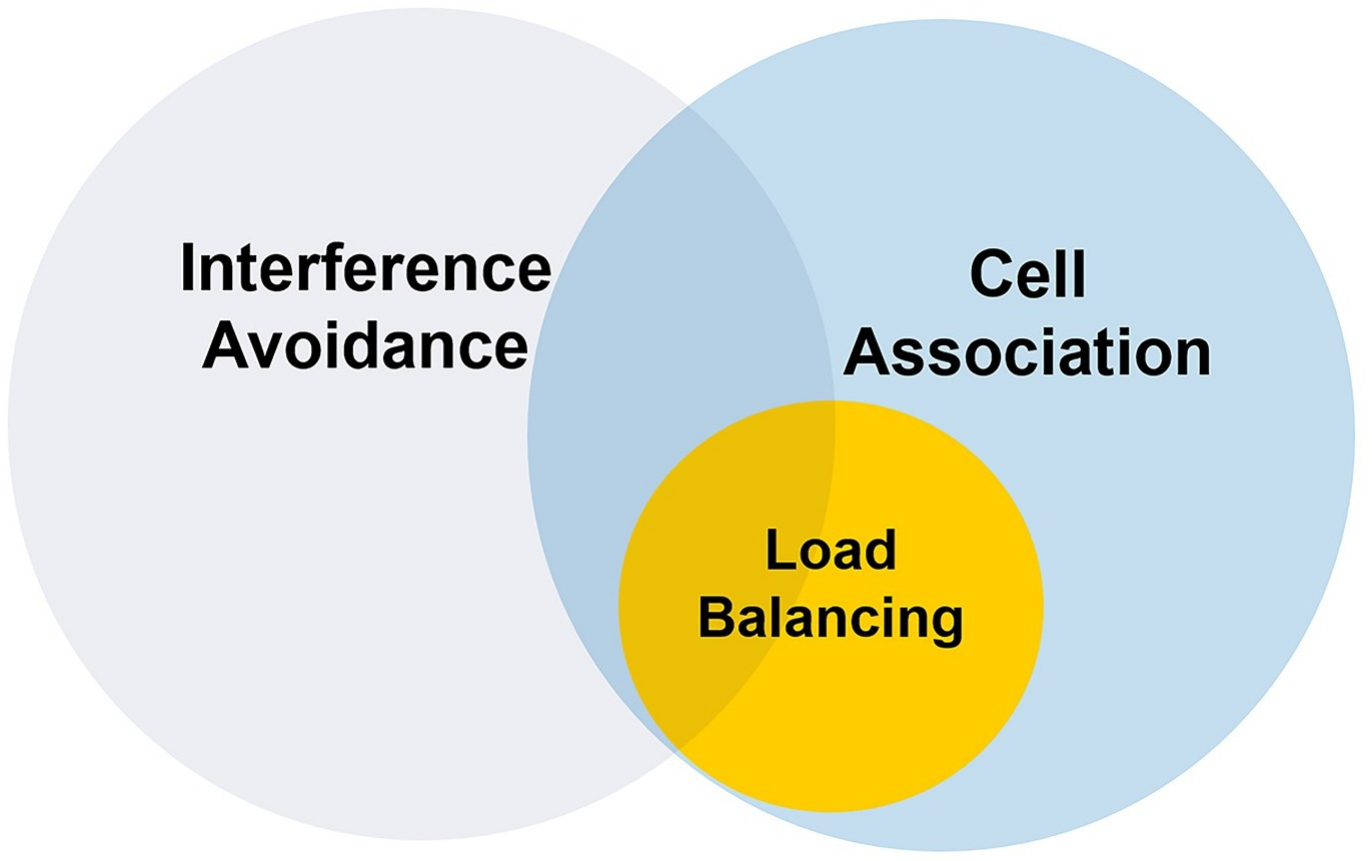
Relationships among cell association, interference avoidance, and load balancing.

Fig 2 illustrates the relationship between the three issues related to HetNets discussed in this paper (i.e., cell association, interference avoidance, and load balancing). In this figure, each circle shows the range of studies performed on each topic in heterogeneous networks. Load balancing is an approach on which cell assignment may or may not be based.

In heterogeneous cellular networks, cell association and interference management are two critical topics that can be handled separately or jointly. In [13], we discussed the necessity of jointly considering these two issues in HetNets. The main reasons for this are summarized below.

- In the cell association process, cells are allocated to users according to their available resources. On the other hand, when interference avoidance techniques are used, cell resources are partitioned among cells, depending on the users assigned to each cell. The answer to each of these problems depends on the answer to the other; therefore, if they are considered together, a better result will be obtained.
- Inappropriate cell assignments in the uplink and downlink can cause intensive interference in the network.
- The process of user offloading to smaller cells with the aim of load balancing and the process of partitioning the inter-cell resources for interference avoidance have mutual effects on network throughput. Thus, these two issues should be considered jointly to increase the network throughput.

A list of related articles on downlink user association and their qualitative comparisons is presented in Table 1.

**Table 1:** Characteristics of the related research on user association, load balancing (LB), and inter-cell resource allocation.

	Ref.	Number of tiers	Backhaul constrained	ICI	Resource used for ICI	Separately /jointly	Centralized /distributed	LB	LB approach	LB optimization problem
**(a) Cell association with load balancing approach**	[42]	two	no	no	-	-	distributed	yes	optimization	Max—(load entropy)
	[43]	two	no	no	-	-	distributed/ centralized	yes	metric-based	-
	[44]	two	no	no	-	-	centralized	yes	metric-based	-
	[38]	multiple	yes	no	-	-	distributed	yes	optimization	Max utility
	[45]	multiple	no	no	-	-	distributed	yes	optimization	Max min utility
	[2]	multiple	no	no	-	-	distributed/ centralized	yes	metric-based /optimization	-
	[21]	multiple	yes	no	-	-	centralized	yes	metric-based	-
	[46]	multiple	yes	no	-	-	distributed	yes	optimization	Max sum log (backhaul load efficiency)
	[14]	multiple	yes	no	-	-	distributed	yes	optimization	Min user service time Min traffic delivery latency
	[18]	multiple	no	no	-	-	centralized	yes	optimization	Min traffic delivery latency
**(b) Cell association with ICI management**	[47]	two	no	yes	power	jointly	distributed	no	-	-
	[48]	multiple	no	yes	time	jointly	distributed	no	-	-
	[39]	two	yes	yes	power	separately	distributed	no	-	-
	[5]	multiple	no	yes	frequency power	jointly	distributed	no	-	-
	[49]	two	no	yes	frequency time	separately	distributed	no	-	-
	[34]	two	no	yes	frequency power	separately	distributed	no	-	-
	[35]	two	no	yes	power	jointly	distributed	no	-	-
**(c) Cell association with load balancing approach with ICI management**	[12]	one	no	yes	frequency	jointly	centralized	yes	optimization	Max sum log (rate)
	[8]	multiple	no	yes	time	separately	distributed	yes	metric-based	-
	[50]	two	no	yes	power	separately	centralized	yes	metric-based/ optimization	-
	[51]	multiple	no	yes	power	jointly	distributed	yes	optimization	Max sum-rate
	[7]	two	no	yes	time	separately	distributed	yes	metric-based	-
	[33]	one	no	yes	time	jointly	distributed	yes	optimization	-
	[23]	one	no	yes	frequency	separately	centralized	yes	optimization	Min load variance
	[24]	multiple	no	yes	frequency	separately	distributed/ centralized	yes	optimization	Max–(load entropy)
	[52]	two	no	yes	frequency	jointly	distributed	yes	optimization	Max sum log (rate)
	[53]	two	no	yes	frequency	separately	distributed	yes	metric-based	-
	[54]	Two	no	yes	frequency	separately	distributed	yes	optimization	Max sum log (rate)
	[55]	two	no	yes	time frequency	separately	distributed	yes	metric-based	-
	[40]	multiple	yes	yes	power (on/off)	jointly	centralized	yes	optimization	Max sum log (backhaul load efficiency)
	[41]	multiple	yes	yes	power	jointly	centralized	yes	optimization	Max min rate
	DB-AC	multiple	yes	yes	time frequency	jointly	distributed	yes	optimization	Max sum log (rate)

The importance of load balancing in HetNets has been noticed in recent years, and many studies have been conducted to solve the cell assignment problem with the load balancing approach, as shown in Table 1, part (a). For the studies in the table which column nine shows the application of load balancing, the ninth and tenth columns are filled that show the load balancing method and the related optimization problem (if any), respectively. However, these articles did not address the issue of ICI.

On the other hand, in Table 1, part (b), there is a list of studies that considered the ICI problem along with the cell allocation problem. As shown in the sixth column, different inter-cell resources have been used to prevent ICI. However, in these studies, the load balance in cell allocation has not been considered.

Several recent studies have addressed this research gap by investigating the two issues in their study, which are shown in Table 1, part (c). In [12, 23, 33], the importance of examining these two issues at the same time network is considered in single-tier. In [34–37], cell assignment and interference management are considered for particular HetNet architectures. For example, in [34], a two-tier network with indoor femto BSs and no inter-cell interference is considered.

In this study, our goal is to provide a practical solution for HetNets with any number of tiers and architectures, which makes it closer to real scenarios. Also, as in [38, 39], the limited backhaul capacity of BSs is considered for load balancing in order to better model the difference between backhaul communication links of BSs in heterogeneous networks.

The authors in [24] investigate load balancing and interference management in multi-tier networks, but the solution of these two combinations is not possible, which leads the system away from optimal resource allocation.

Works [40] and [41] addressed the joint problem of ICI and LB in the CDMA-based network with diverse QoS requirements of users, but with the assumption of the existence of a central point, they proposed a centralized solution, whereas distributed solutions are more practical in HetNets, especially with dense deployment of small cells.

This paper aims to address the aforementioned gaps by proposing a distributed comprehensive solution for the joint allocation of cells and inter-cell resources, which balances the load among cells and protects all users prone to interference from all tiers against intensive interference in backhaul-constrained multi-tier networks.

## 3. System model and problem formulation

### 3.1. System model

In this study, a downlink HetNet with *K* tiers (*K* = 1, 2, …) is considered, where each tier models a particular type of BS. Let *B* and *U* be the total number of BSs and users, respectively, where B and U are the sets of all BSs and all users in the network, respectively.

The heterogeneity of the network, which is basically the differences among BSs and the difference between the tiers, is modeled using different values of parameters such as the maximum power and backhaul connection capacity of BSs and the differences in the spatial densities of BSs in different tiers. The transmission power of each BS *j* on each subband *m* has two states. In the case of connection, it has the constant power of *P*_*jm*_; otherwise, it is 0.

The BSs are supposed to be in a co-channel deployment; therefore, the available bandwidth is shared among cells and is divided into *M* subbands with an equal bandwidth *W*. Each subband includes multiple subcarriers and is a frequency unit within which transmissions can be coordinated among BSs. The set M is considered to be the set of all subbands.

The OFMDA method is used so that each subband in each cell is assigned to only one user. BSs have no restrictions on users, and in fact, all operate in the open access mode. We assume that each user can connect to only one BS at a time. It is also assumed that a maximum of one subband can be assigned to each user.

The assignment identifiers $\left\{x_{ji}^{m}\right\}$ represent the cell assignment to each user on each subband. In this way, whenever user *i* is connected to BS *j* on subband *m*, $x_{ji}^{m}=1$; otherwise $x_{ji}^{m}=0$. Generally, the SINR of each connection is denoted by $\gamma_{ji}^{m}$ and is presented in the following form:

$$\gamma_{ji}^{m}=\frac{P_{jm}h_{ji}}{\sum_{k\in B-\left\{j\right\}}P_{km}h_{ki}+\sigma^{2}}$$ (1)

where *P*_*jm*_ is the transmission power of BS *j* on subband *m*, *h*_*ji*_ shows the channel gain between BS *j* and user *i*, and *σ*^2^ is the additive white Gaussian noise power at users. Given that interfering connections are avoided in the interference model of this study (discussed in subsection 4.2) by managing the allocation of subbands in adjacent cells, the denominator of (1) is reduced to *σ*^2^. If user *i* is connected to BS *j* on subband *m*, its achievable data rate, according to Shannon’s formula, is as follows:

$$R_{ji}^{m}=W\;{\text{log}}_{2}\left(1+\gamma_{ji}^{m}\right)$$ (2)

According to the system model, in each time interval with constant values of channel gain, $R_{ji}^{m}$ has a fixed value. So the total rate of user *i* is represented by *R*_*i*_ and is as follows.


$$R_{i}=\underset{j\in B}{\sum }\;\underset{m\in M}{\sum }x_{ji}^{m}R_{ji}^{m}$$ (3)

### 3.2. Interference model

In this study, the protocol interference model is used [56, 57]. Owing to the use of OFDMA technology and its subnet allocation rules—as well as the purpose of this study, which is to prevent interference for all active connections—this interference model fits well with the system model under study. The protocol interference model also helps reduce the complexity of the problem. This model of interference for cellular networks is described below, and some required definitions are presented.

The set of BSs whose transmitted signals are received by user *i* (and which can actually have data transmission or interference for user *i*) is called the user *i* interference set and is represented as ${BI}_{i}$. On the other hand, the set of users receiving the signal of BS *j* is denoted by ${UI}_{j}$ and is called the BS *j* interference set.

In this interference model, two links interfere if the transmitter of each link is a member of the interference set of the other link receiver. More precisely, (*j*, *i*) and (*k*, *z*) interfere if $j\in {BI}_{z}$ or $k\in {BI}_{i}$. According to the interference model, for the transmission of BS *j* to user *i* on subband *m* in a time interval, other interfering links need not be connected, which is done by turning off the transmission of interfering BSs (i.e., $k\in {BI}_{i}$) on subband *m*.

For example, in Fig 1, Link (*BS*3, *d*) and link (*BS*1, *a*) do not interfere with each other. Therefore, they have the possibility of simultaneous connection on one subband. However, link (*BS*3, *d*) interferes with (*BS*1, *b*) and (*BS*2, *c*). If one of these links is transmitting on subband *m*, the other two links should not be active on this subband.

The assignment array **x** contains all assignment variables $x_{ji}^{m}$ in the system. Any system model that considers $x_{ji}^{m}$ as a binary variable is called a discrete system. By linearly relaxing the assignment variables, the identifier $x_{ji}^{m}$ takes a continuous value in the interval [0, 1]. In this case, the generated system model is called a continuous system. The variable $x_{ji}^{m}$ in the continuous system represents the time percentage of the connection of BS *j* to user *i* on subband *m*.

An assignment array in a discrete system is feasible whenever connected links can transmit data simultaneously without interference. In other words, no two connections interfere with each other. An assignment array in a continuous system is feasible whenever a central scheduler with access to all network information can schedule all network links to achieve the percentage of their connection time without interference [58].

### 3.3. Problem formulation

In this section, the problem of our study is formulated as a mathematical optimization problem for cell and subband assignment.

$$P1:\;\underset{x}{\text{max}}\;\underset{i\in U}{\sum }\;U_{i}\left(\underset{j\in B}{\sum }\;\underset{m\in M}{\sum }\;x_{ji}^{m}R_{ji}^{m}\right)$$ (4)

subject to $x_{ji}^{m}\in \left\{0,1\right\}$

$$\underset{i\in U}{\sum }\;x_{ji}^{m}\leq 1,\;\;\forall j\in B,m\in M$$ (5)

$$\underset{j\in B}{\sum }\;\underset{m\in M}{\sum }\;x_{ji}^{m}\leq 1,\;\;\forall i\in U$$ (6)

$$x_{ji}^{m}=0,\;\;\;\forall i\in U,m\in M,\;j\in B-BI_{i}$$ (7)

$$\underset{i\in U}{\sum }\;\underset{m\in M}{\sum }\;x_{ji}^{m}R_{ji}^{m}\leq C_{j},\;\;\;\forall j\in B$$ (8)

$$\underset{i\in UI_{j}}{\sum }\;x_{ji}^{m}+\underset{i\in UI_{j,k}}{\sum }\;x_{ki}^{m}\leq 1,\;\;\forall m\in M,j\in B,k\in B-\left\{j\right\}$$ (9)

where ${UI}_{j,k}={UI}_{j}\cap {UI}_{k}$. In this problem, the objective function is to maximize the network utility, which is the total utility of the users. The utility function is defined in such a way that network utility maximization, considering the constraints, leads to an improvement in network throughput while balancing the load for better utilization of small cells. Constraint (5) is set according to the OFDMA technology, which states that each subband in each cell can be used by only one user. Constraint (6) applies a single connection so that each user can connect to no more than one cell and one subband. Constraint (7) is applied so that users connect only to the BSs of their interference set. The backhaul capacity constraint is applied by constraint (8), where *C*_*j*_ is the maximum total rate that BS *j* can provide according to its backhaul connection network.

The last constraint in P1 is called the interference constraint. Due to the interference model used in this research, it is necessary to impose an interference constraint in the optimization problem to prevent interfering connections. Several interference constraints have been proposed in the literature [57], which can be used for this purpose. This choice varies for each problem since the formation of the interference constraint greatly affects the complexity of the problem, how the problem is decomposed, and the final algorithm in terms of computational complexity and signaling.

In this study, the interference constraint of two BSs [57] is selected, as it provides the necessary and sufficient condition for the feasibility of the assignment array in our discrete system model. This constraint makes some problem simplifications possible and can lead to a well-distributed algorithm.

The user *i* utility function, *U*_*i*_, is considered a continuously differentiable and strictly concave function [12, 26]. Thus, the objective function of the problem, which is the sum of *U*_*i*_ of users, is also differentiable and strictly concave. In this study, we select the logarithmic utility function that would also lead to load balancing in the network. The logarithmic function is concave and therefore has diminishing returns, giving rise to load balancing consistent with the philosophy of resource allocation in real systems. That is, allocating more resources to already well-served users has a lower priority. In contrast, it is desirable to provide resources for users at lower rates (i.e., in the lower part of the logarithm function) [19].

If *R*_*i*_ represents the user *i* achievable data rate, the utility function in this paper, as in [59], is defined as *U*_*i*_ (*R*_*i*_) = log(1 + *R*_*i*_). This function has all the features expected from the logarithm function. In addition, adding a positive value to the user rate before applying the logarithm function causes the value of its utility function to become zero instead of an undefined value if the a user rate is zero. For any case where the user rate value is not zero, with respect to *R*_*i*_ ≫ 0, log(1 + *R*_*i*_) ≅ log(*R*_*i*_) is established [60]. The addition of one also facilitates problem-solving in the remainder of this study. Using this utility function, the objective function of the problem takes the form of function (10).


$$\underset{i}{\sum }\;U_{i}\left(R_{i}\right)=\underset{i}{\sum }\;\text{log}\left(1+R_{i}\right)=\underset{i}{\sum }\;\text{log}\left(1+\underset{j}{\sum }\;\underset{m}{\sum }\;x_{ji}^{m}R_{ji}^{m}\right).$$ (10)

Problem P1, which can be classified as a generalized assignment problem, is an integer nonlinear program, making it NP-hard. This problem is discussed in continuous and discrete systems in the following sections.

## 4. Problem-solving

### 4.1. Centralized gradual removal algorithm using the time-sharing mode

Assuming that BSs and user equipment can work in both exclusive and time-sharing modes, the resource allocation problem can be formulated into two modes. In P1, cell and subband allocations are formulated for the exclusive state. In exclusive mode, each subband of a BS can be used by only one user in a time frame, whereas in time-sharing mode, multiple users can share a subband within a time frame [34, 61]. In the time-sharing mode, assuming that the BSs can be switched rapidly, assignment variables have continuous values in the interval [0,1]. In this case, the system is continuous, and variable $x_{ji}^{m}$ represents the time percentage of the connection of BS *j* to user *i* on the subband *m* [62]. The optimization problem P2 illustrates the problem of cell and subband allocation in the continuous system.

$$P2:\underset{x}{\text{max}}\;\underset{i\in U}{\sum }\;U_{i}\left(\underset{j\in B}{\sum }\;\underset{m\in M}{\sum }\;x_{ji}^{m}R_{ji}^{m}\right)$$

subject to **$x_{ji}^{m}\in [0,1]$**

(5), (6), (7), (8)

$$\underset{\left(j,i\right)\in Q_{n}}{\sum }\;x_{ji}^{m}\leq \beta ,\;\;\forall Q_{n}\in \Phi ,\;\forall m$$ (11)

where *Φ* is the set of all maximal cliques of the interference graph of the considered network. As in [58], the concept of an “interference graph” is used in this study. This graph shows the interference between different network links. The interference graph is unidirectional, and each node is equivalent to a possible connection in the wireless network. An edge exists between two nodes if the two equivalent wireless connections interfere with each other according to the interference model [57].

In P2, unlike P1, another interference constraint—called scaled maximal clique—is used in (11) [57]. This form of constraint is appropriate for the time-sharing mode since with the proper selection of *β* (where 0 < *β* ≤ 1), it provides sufficient conditions and falls within a fixed range of necessary conditions for the feasibility of an assignment array in a continuous system [63].

P2 is a convex optimization problem. Therefore, its global optimal answer can be easily obtained from existing solutions for convex problems, such as interior-point methods [64]. In fact, the convex problem solution provides a centralized solution to the cell allocation and subband allocation problems in time-sharing mode. Furthermore, solving problem P2 provides an upper bound to the exclusive mode problem P1.

By solving problem P2 and applying the technique of gradual removal [65], a solution for the exclusive mode problem can be obtained. In Algorithm 1, the gradual removal approach algorithm (called GR-AC) is presented; this algorithm is utilized to find binary associations for cell and subband allocation.

**Algorithm 1** Algorithm of Gradual Removal for the Association of Cell and Subband (GR-AC)

1: **Initialization**: *β* ← 1, *continue* ← true.

2:  **While** (*continue* = true)

3:  solve problem P2

4:  **if** all the variables $x_{ji}^{m}\in \{0,1\}$ **then**

5:   *continue* ← false

6:  **else**

7:   $\left(j^{*},i^{*},m^{*}\right)\leftarrow arg\;{\text{min}}_{\left(j,i,m\right)}\left[x_{ji}^{m}\;\right]$ such that $x_{ji}^{m}\neq 0$

8:   $x_{j^{*}i^{*}}^{m^{*}}\leftarrow 0$

9:  **end if**

10:  **end**

The GR-AC algorithm is an iterative algorithm that solves the convex problem P2 and in each iteration sets the value of the least non-zero assignment $x_{ji}^{m}$ to zero. This algorithm concludes when all variables $x_{ji}^{m}$ become binary. The maximum number of iterations in the GR-AC algorithm is *O*(*B* × *U* × *M*), where *B*, *U*, and *M* are the sizes of the sets of BSs, users, and subbands, respectively.

Note that the value of the parameter *β* of problem P2 in this algorithm must be equal to one. This is because it seeks an answer for the discrete system model, and the interference constraint of the maximal clique (without any scale) provides the necessary and sufficient condition for the feasibility of the assignment array in the discrete system model [57].

In fact, the GR-AC algorithm is a heuristic algorithm that solves the P1 problem by providing suboptimal answers. In subsection 5.2, the simulation results of this algorithm are examined. Due to the use of the maximal clique interference constraint, the GR-AC algorithm cannot be used for a high-mobility network unless the maximal cliques of the interference graph are found at each period of the algorithm execution, which, in turn, increases the temporal and computational complexity of the network. Furthermore, the implementation of this algorithm requires the use of a central node that has complete information about the channel status and the interference set of the BSs. Meanwhile, centralized solutions are impractical in HetNets (especially with dense deployment). Therefore, it is still necessary to find a distributed and efficient algorithm for resource allocation and interference management that can be implemented in practice.

### 4.2. Cell and subband association in the exclusive mode

In this section, we will address problem P1 again and make it tractable through reformulation. In P1, the OFDMA constraint (5) and the interference constraint (9) are used. In multicellular networks, the interference constraint covers the OFDMA constraint; thus, by assuming that the network is multicellular, the OFDMA constraint is omitted. Furthermore, the objective function in P1 can be simplified according to the following proposition.

**Proposition**. In the discrete system model, objective function (10) is equivalent to expression (12).

$$\underset{i}{\sum }\;U_{i}\left(R_{i}\right)=\underset{i}{\sum }\;\underset{j}{\sum }\;\underset{m}{\sum }\;x_{ji}^{m}\;\text{log}\left(1+R_{ji}^{m}\right).$$ (12)

**Proof**. When computing user *i* data rate, the value of $x_{ji}^{m}$ can be set to one for no more than one BS and one subband. Therefore, two states can be considered for each user *i*. The first state is that a connection is established for this user and the value of $x_{j^{\prime }i}^{m^{\prime }}=1$; the second state is that this user is not connected to any BS or subband. In the first case, the utility of user *i* is the same, $\text{log}\left(1+R_{j^{\prime }i}^{m^{\prime }}\right)$, in Eqs (10) and (12). In the second case, the utility of user *i* in (10) and (12) are both zero. Therefore, Eqs (10) and (12), which are the total utility of network users, are equivalent. ■

Problem P3 is equivalent to problem P1 in a discrete and multicellular system. Problem P3 is integer linear programming.

$$P3:\;\underset{x}{\text{max}}\;\underset{i}{\sum }\;\underset{j}{\sum }\;\underset{m}{\sum }\;x_{ji}^{m}\;\text{log}\left(1+R_{ji}^{m}\right)$$

sunject to $x_{ji}^{m}\in \{0,1\}$

(6), (7), (8), (9)

Lagrange dual decomposition [66] is used to design a distributed algorithm for problem P3, which is similar to the method used in [2, 19]. For this purpose, an LP problem called P4 is first obtained via the linear relaxation of P3.

The dual decomposition method provides an appropriate answer when there is a strong duality condition for the problem. There is a strong duality condition for problem P4 with respect to the Slater condition [64] because the objective function and all constraints are linear functions of **x**, and this problem is always feasible (e.g., **x** = 0 is always a feasible point).

In problem P4, there are two coupling constraints, which are the interference constraint and the backhaul constraint. By adding the Lagrange multipliers ***λ*** and ***ν***, these coupling constraints are relaxed and added to the objective function. The Lagrangian function L(x,λ,ν) is obtained according to Eq (13).


$$\begin{array}{ll} L\left(x,\lambda ,\nu \right) & =\sum_{i\in U}\;\sum_{j\in B}\;\sum_{m\in M}x_{ji}^{m}\;\text{log}\left(1+R_{ji}^{m}\right)\\ & -\sum_{j\in B\;}\;\sum_{k\in B-\left\{j\right\}\;}\;\sum_{m\in M}\;\lambda_{jkm}\left(\sum_{i\in UI_{j}}\;x_{ji}^{m}+\sum_{i\in UI_{j,k}}\;x_{ki}^{m}-1\right)\\ & -\sum_{j\in B\;}\;\nu_{j}\left(\sum_{i\in U}\;\sum_{m\in M}\;x_{ji}^{m}R_{ji}^{m}-{\text{C}}_{j}\right). \end{array}$$ (13)

The expression (13) can be decomposed according to index *i*. Therefore, by decomposing it on the basis of *i*, the Lagrangian function for each user *i* is as follows:

$$\begin{array}{ll} L_{i}\left(x_{i},\lambda ,\nu \right) & =\sum_{j\in B}\;\sum_{m\in M}\;x_{ji}^{m}\;\text{log}\left(1+R_{ji}^{m}\right)-\sum_{j\in BI_{i}}\;\sum_{k\in B-\left\{j\right\}}\;\sum_{m\in M}\;x_{ji}^{m}\lambda_{jkm}\\ & -\sum_{j\in BI_{i}}\;\sum_{k\in BI_{i}-\left\{j\right\}}\;\sum_{m\in M}\;x_{ki}^{m}\lambda_{jkm}-\sum_{j\in B}\;\sum_{m\in M}\;\nu_{j}x_{ji}^{m}R_{ji}^{m}. \end{array}$$ (14)

After renaming the indexes in the third expression (14), the sub-problem P_*i*_ for each user *i* finally becomes in the following form:

$${\text{P}}_{i}:\;g_{i}\left(\mu ,\nu \right)=\;\underset{x}{\text{max}}\;\underset{j\in BI_{i}}{\sum }\;\underset{m\in M}{\sum }\;x_{ji}^{m}\left[\text{log}\left(1+R_{ji}^{m}\right)-\underset{k\in B-\left\{j\right\}}{\sum }\;\lambda_{jkm}-\underset{k\in BI_{i}-\left\{j\right\}}{\sum }\;\lambda_{kjm}-\nu_{j}R_{ji}^{m}\right]$$

subject to $x_{ji}^{m}\in [0,1]$

$$\underset{j\in B}{\sum }\;\underset{m\in M}{\sum }\;x_{ji}^{m}\leq 1$$

$$x_{ji}^{m}=0,\;\;\;\forall \;m,\;j\notin BI_{i}$$

The expression multiplied by the variable $x_{ji}^{m}$ in the objective function of P_*i*_ is called the qualification factor of BS *j* and subband *m* from the user *i*’s viewpoint and is denoted by F_*jim*_.


$${\text{F}}_{jim}=\text{log}\left(1+R_{ji}^{m}\right)-\underset{k\in B-\left\{j\right\}}{\sum }\;\lambda_{jkm}-\underset{k\in BI_{i}-\left\{j\right\}}{\sum }\;\lambda_{kjm}-\nu_{j}R_{ji}^{m}.$$ (15)

Higher F_*jim*_ value for user *i* can be achieved through a higher data rate (higher $R_{ji}^{m}$) for the connection, subband *m* not being assigned to other users by BS *j* and other BSs in its interference area (lower relevant multipliers ***λ***), or a less occupied backhaul network of BS *j* with (lower relevant multipliers ***ν***).

For each user *i*, the objective function in P_i_ is a weighted average of the qualification factors corresponding to *i*. Thus, the coefficient of each lies between 0 and 1, and the sum of the coefficients is equal to one. If the maximum value of F_*jim*_ occurs for only one BS and subband pair (j*, m*) for each user *i*, the unique response of P_i_ is obtained by holding this maximum value F_*jim*_ for the pair (j*, m*) (i.e., $x_{j^{*}i}^{m^{*}}=1$) and reducing the effect of other elements (i.e. $x_{ji}^{m}=0\;for\;\left(j,m\right)\neq \left(j^{*},m^{*}\right)$).

As a result, when solving P_i_, after receiving information from the environment, each user simply needs to choose a pair (*j*, *m*) that satisfies two conditions: the BS *j* must be within the user interference set and maximize the expression F_*jim*_ for that user. In case (15) has more than one maximizing answer, the user chooses one randomly.

With the relaxation of the interference and backhaul constraints in P4, this problem transforms into two levels of optimization problems. At the lower level, there are sub-problems with one P_i_ sub-problem for each user *i*. At the higher level, a dual master problem is responsible for updating the dual variables ***λ*** and ***ν*** by solving the dual master problem (16).


$${\text{P}}_{\text{D}}:\;\underset{\lambda ,\nu \geq 0}{\text{min}}\;g\left(\lambda ,\nu \right)=\underset{i\in U}{\sum }\;g_{i}\left(\lambda ,\nu \right)+\underset{j\in B\;}{\sum }\;\underset{k\in B-\left\{j\right\}}{\sum }\;\underset{m\in M}{\sum }\;\lambda_{jkm}+\underset{j\in B\;}{\sum }\;\nu_{j}{\text{C}}_{j}.$$ (16)

The dual function *g*_*i*_ (***λ***, ***ν***) is derived from maximizing P_i_ with the given values of ***λ*** and ***ν***. The dual master problem in P_D_ can be solved following the subgradient method and the Jacobi algorithm [60, 66]. Accordingly, each BS *j* updates *ν*_*j*_ and *λ*_*jkm*_ for each of its neighboring BSs *k* values based on the following expressions:

$$\lambda_{jkm}\left(t+1\right)={\left[\lambda_{jkm}\left(t\right)+\beta \left(t\right)\left(\underset{i\in UI_{j}}{\sum }\;x_{ji}^{m}+\underset{i\in UI_{j,k}}{\sum }\;x_{ki}^{m}-1\right)\right]}^{+}.$$ (17)

$$\nu_{j}\left(t+1\right)={\left[\nu_{j}\left(t\right)+\beta \left(t\right)\left(\underset{i\in U}{\sum }\;\underset{m\in M}{\sum }x_{ji}^{m}R_{ji}^{m}-{\text{C}}_{j}\right)\right]}^{+}.$$ (18)

The operator [.]^+^ is applied to the multipliers because the Lagrangian multipliers have non-negative values [64]. *t* is the iteration index of the algorithm, and *β*(*t*) is a positive step size with two conditions $\sum_{t=1}^{\infty }\beta \left(t\right)=\infty$ and lim_*t*→∞_ *β*(*t*) = 0.

If *β*(*t*) meets the above conditions, the convergence of the descending subgradient method is guaranteed, assuming that the assignment variables are continuous in the range [0,1] [67]. These conditions do not guarantee the convergence of the binary allocation variables. Nevertheless, *β*(*t*) = 0.5⁄*t* is used in the simulations of this study.

In this method, the values of the assignment variables and the Lagrange multipliers are iteratively updated to achieve convergence. Note that the updating rule for assignment variables automatically generates binary variables, meaning that no further approximation is required.

### 4.3. Feasibility problem and admission control

In the cell allocation process described in the previous subsection, each user finds its desired BS and subband for making a connection request. Users are unaware of any other requests made to that BS and the resources allocated to each BS. Therefore, multiple users may request a specific resource simultaneously, which violates backhaul constraint (8) and interference constraints (9). These concurrent requests not only disturb the convergence of the algorithm, but also provide an infeasible answer to the problem [64]. Therefore, if the answer obtained in an iteration is beyond the feasible range, it is necessary to project it to the feasible set.

The use of heuristic methods have been suggested in the literature to generate feasible responses from intermediate or final answers when combining the dual decomposition method with the subgradient method for linear integer problems [2, 68]. This heuristic method requires the addition of two functions to our solution. First, each user *i* needs to sort the BS and subband pairs in descending order based on the qualification factor obtained in (15) during the BS selection step; also, while sending the request to the BS j*, this sorted list (called the user *i* selection list) is also sent.

After receiving users’ requests by BSs, user admission should be performed based on the backhaul and interference constraints. For this purpose, the BS uses a parameter called the desirability value for each of its connections. This parameter can be defined in different ways. In subsection 5.3.1, its different definitions are examined and compared.

A BS executes an algorithm called CNR (shown in Algorithm 2) for any received connection request. The request can be accepted if its selection maintains the network feasibility status and improves the network utility. In the CNR algorithm, the backhaul and interference constraints must be checked for the new request, resulting in one of the two resulting outcomes:

- The user’s request will not be accepted, and the user will be referred to its next choice. Therefore, at this stage, the network remains in its current state.
- The request of user *i* is approved resulting in the refusal of the previous request accepted in (*j*, *m*) and interfering requests from nearby BSs. These refused users will then be referred to their next selections.

**Algorithm 2** Algorithm of Checking New Request (CNR) for each request (***j***, ***i***, ***m***).

1: **Initialization**: *sumDes* ← 0, *interfereList* ← ∅, *Capacity* ← true.

2: **for** k∈B **repeat**

3:  *z* ← the user that has been associated with BS *k* and subband *m*

4:  **If** *z* ≠ ∅ **and** $\left(j\in {BI}_{z}\;or\;k\in {BI}_{i}\right)$ **then**

5:   *sumDes* ← *sumDes* + *desirability*(*k*, *z*, *m*)

6:   *interfereList* ← *interfereList* ∪ (*k*,*z*,*m*)

7:   **If** *k* = *j* **then $c=R_{jz}^{m}$ end if**

8:  **end if**

9: **end for**

10: **If** (*desirability* (*j*, *i*, *m*) > *sumDes*) **and** (*occupied capacity of BS j untill now* − $c+R_{ji}^{m}<C_{j}$) **then**

11:  associate user *i* to BS *j* and subband *m*

12:  **for** (*k*, *z*, *m*) ∈ *interfereList* **repeat**

13:   unassociate user *z* to BS *k* and subband *m*

14:   (*k*′, *m*′) ← the next selection in user *z* list

15:   *CNR*(*k*′, *z*, *m*′)    //refer user z to the next BS

16:  **end for**

17: **else**

18:  (*j*′, *m*′) ← the next selection in user *i* list

19:  *CNR*(*j*′, *i*, *m*′)   //refer user i to the next BS

20: **end if**

Each request triggers the execution of the algorithm, and this process continues for each user until it is accepted in one of its selections or the user selection list has finished being checked (and, as a result, that user is not accepted). This process yields a response in the feasible set.

It is assumed that the BSs are connected by high-speed backhaul networks. Thus, the required message exchange takes place in a short period. Additionally, the admission control outlined in this section aids in load balancing the network by preventing congestion of users connected to a BS.

The size of each user’s selection list (represented by *R*) determines the maximum number of selections (*j*, *m*) that will be checked. This parameter can be limited to reduce the time complexity of the algorithm. Subsection 5.3.2 examines the effect of selection list size on algorithm performance.

### 4.4. Distributed joint cell and subband allocation

The proposed algorithm for cell and subband allocation based on dual decomposition (discussed in the previous sections) is called the DB-AC algorithm, which is shown in Algorithm 3.

**Algorithm 3** Algorithm of Dual-based Association of Cell and Subband (DB-AC).

1: **Initialization**: ***λ*** ← **0, *ν*** ← **0** and *t* ← 0.

2: **repeat**

3:  *t* ← *t* + 1

4:  **for** i∈U **repeat**

5:   **for** $j\in {BI}_{i},\;m\in M$ **repeat**

6:    Calculate F_jim_ with (15)

7:   **end for**

8:   *Req*_*i*_ ← the first *R* pair of (*j*, *m*) in descending order of F_*jim*_

9:   (j*, m*) ← arg max_(*j*,*m*)_ [F_*jim*_]

10:   *CNR*(j*, *i*, m*) //refer user *i* to its first selection

11:  **end for**

12:  **for** j∈B **repeat**

13:   Calculate *ν*_*j*_ (*t* + *1*) with (18)

14:   **for** k∈B,m∈M **repeat**

15:    Calculate *λ*_*jkm*_ (*t* + *1*) with (17)

16:   **end for**

17:  **end for**

18: **until** convergence or *t* = *T*_*max*_

One of the advantages of the proposed algorithm is that it uses local information. This way, each user only needs the information broadcast by the BSs within its interference area. Moreover, each BS is required to exchange information only with its surrounding BSs. This feature, along with the local computations of each network element, eliminates the need for the algorithm to have a central node, enabling its practical implementation in new-generation networks.

In each iteration of the DB-AC algorithm, each user must calculate the qualification factor for the BSs in its interference area and all subbands and sort the first *R* selections according to the qualification factor. The algorithm complexity for each user is directly influenced by the complexity of the sorting algorithm. In the worst case, the interference set of a user includes all network BSs. Therefore, the time complexity of the algorithm for each user in each iteration is *O*(*B* × *M* × *R*).

On the other hand, each BS receives the user’s request in each iteration and performs user admission control (line 10 in DB-AC). In the worst case scenario, all users’ selection lists are checked. Therefore, the CNR algorithm is called *O*(*U* × *R*) times. In each call, the relevant request is compared with the interfering requests of neighboring BSs. The number of neighboring BSs in the worst case includes all network BSs. Therefore, the time complexity of the algorithm for all BSs in this step is equal to *O*(*U* × *R* × *B*). Then, based on the obtained response, each BS updates its Lagrangian multipliers, the number of which is equal to the number of BSs around it, multiplied by the number of subbands (line 15 in DB-AC). Therefore, the algorithm’s time complexity for each BS in this step of algorithm iteration is *O*(*B* × *M*). Assuming that the algorithm requires T iterations, its total time complexity is *O*(*T*(*B* × *U* × *M* × *R*)). If T is not of the order *B*, *U*, *M*, and *R*, as the simulations show, the complexity of the algorithm is equal to *O*(*B* × *U* × *M* × *R*).

Depending on the rate of changes in the network, the proposed algorithm can allocate resources either in each subframe or over a longer period consisting of several subframes. An alternative is to employ triggers as indicators of network changes and, therefore, the need for re-running the algorithm.

## 5. Simulation and performance evaluation

This section starts with the presentation of the general simulation conditions. Then, the evaluation of the work has been conducted done in three subsections. In the first part, considering that the two proposed algorithms, GR-AC and DB-AC, are obtained from some optimization problems, their results and execution times are compared with those of solving the optimization problems. Following that, we focus is on evaluating the distributed DB-AC algorithm. In the second part, the effect of some important parameters on the algorithm is examined. The third part covers the comparison of the DB-AC algorithm with the baseline approaches under various network conditions.

### 5.1. Simulation setup

In the simulations, heterogeneous networks consisting of three tiers (macro, pico, and femto) are considered. A square area of 1000 m × 1000 m is considered that the macro BS remains at the center in the middle of this space. The locations of other BSs and users are determined randomly with a uniform distribution.

The average transmission powers of macro, pico, and femto BSs are 40, 1, and 0.1 W, respectively, but the transmission powers in different subbands vary around the average value by 2, 0.2, and 0.02 W, respectively. The noise power is assumed to be -100 dBm for all receivers.

As in the related literature [69], the common function $h_{ji}=kd_{ji}^{\alpha }$ is used to model the path loss, where *d*_*ji*_ is the distance between BS *j* and user *i*. The parameters *k* and *α* are the path loss coefficient and exponent, which are considered to be 0.9 and -3, respectively. The bandwidth of each subband is 180 kHz (i.e., the bandwidth of a resource block in LTE standard).

According to the interference model, it is necessary to define the interference set of each user. In order to determine the interference area of each user, a parameter called interference range power limit (IRPL) is defined. This parameter indicates the minimum received power of a BS signal at a user that qualifies the BS as a member of that user’s interference set. Usually the physical layer technology in use determines this threshold. In the simulations in this section, the IRPL value for all connections is 1e-8 W.

The desirability function is considered ${des}_{ji}^{m}=\text{log}\left(R_{ji}^{m}+1\right)$. However, in Section 5.3.1, different desirability functions are examined. The effect of different values on the backhaul connection capacity of the BSs is investigated in section 5.3.3. In other sections, the BSs do not have a limit on their backhaul connection capacity. In this study, MATLAB R2017A software is used to implement and simulate the proposed algorithms.

### 5.2. Comparison between the presented approaches

In this section, we explore four different approaches discussed earlier to solve the cell and subband allocation problem: solving the time-sharing mode problem P2, solving the exclusive mode problem P3, utilizing the GR-AC algorithm, and utilizing the DB-AC algorithm. The CVX toolbox in the MATLAB environment [70] has been used to gain optimal results for P2. Problem P3 is linear integer programming, which is solved using the MATLAB optimization toolbox.

This section aims to compare the performance of GR-AC and DB-AC algorithms with each other and their respective optimization problems. In addition the outcomes of time-sharing mode and exclusive mode are analyzed.

**Fig 3:**
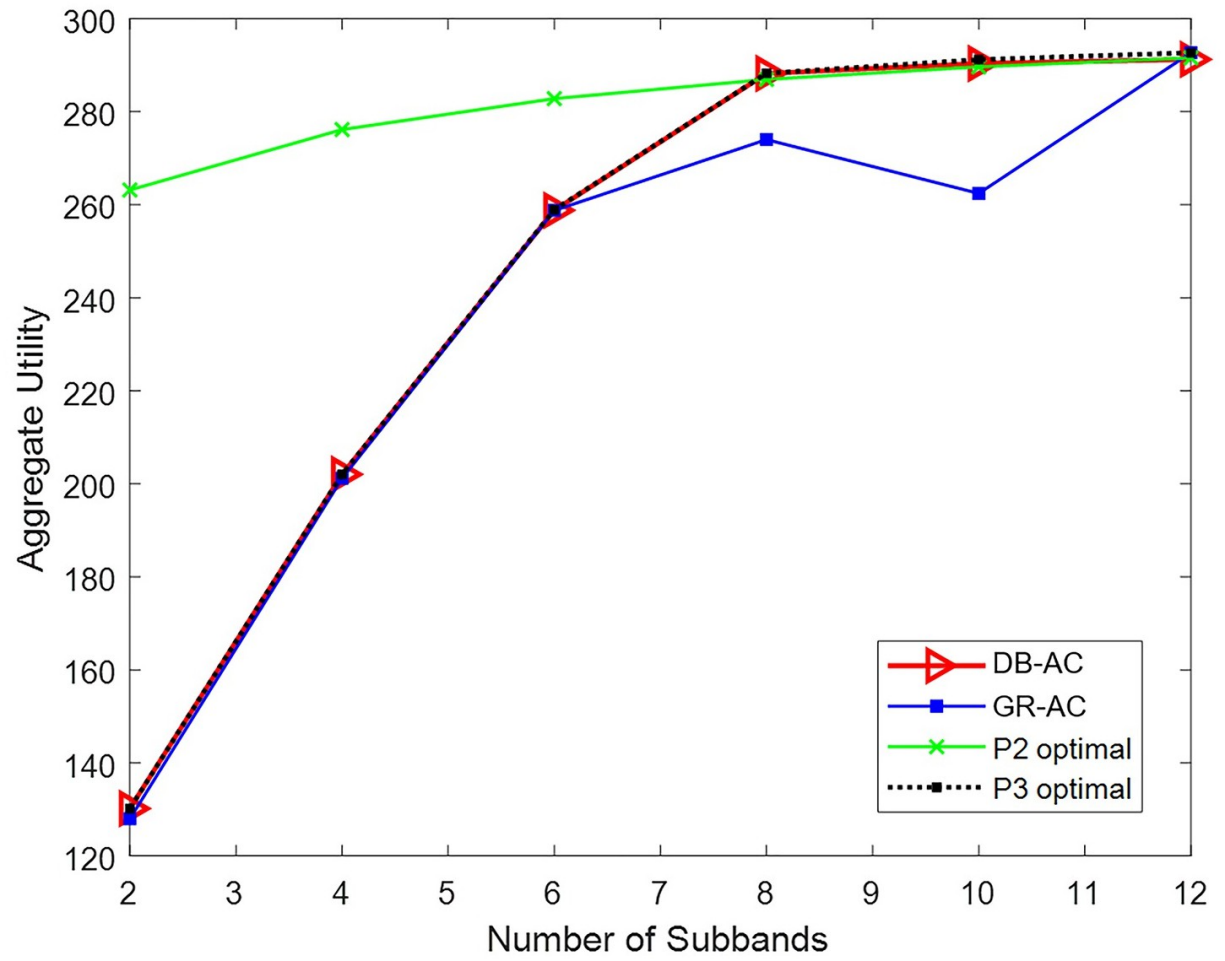
Result of the objective function for four problem answers for different numbers of subbands.

A scenario with one macro BS, two pico BSs, and four femto BSs, shown as (1,2,4) BSs, is considered. Twenty users are distributed in the network, and the number of subbands for each BS increases from two to twelve. In problem P2, the value of *β* is assumed to be 0.86. In the DB-AC algorithm, the size of the selection list is assumed to be 50. Fig 3 shows the value of the objective function for the four methods used to solve the cell and subband allocation problem.

As seen in Fig 3, the result of the proposed DB-AC algorithm overlaps the optimal answer to P3 to a great extent. The optimal answer for problem P2 is, in fact, the optimal answer for the time-sharing mode problem. Therefore, its network utility is an upper bound for the results of other methods. The proposed GR-AC algorithm provides a suboptimal answer to the problem. With the advancement of network subbands—and, therefore, network capacity, the need for resource time-sharing decreases. Increasing the number of subbands in Fig 3 brings the P3 exclusive mode answer closer to the P2 time-sharing mode answer and causes overlap at eight subbands due to active connections of all users being in exclusive mode.

**Fig 4:**
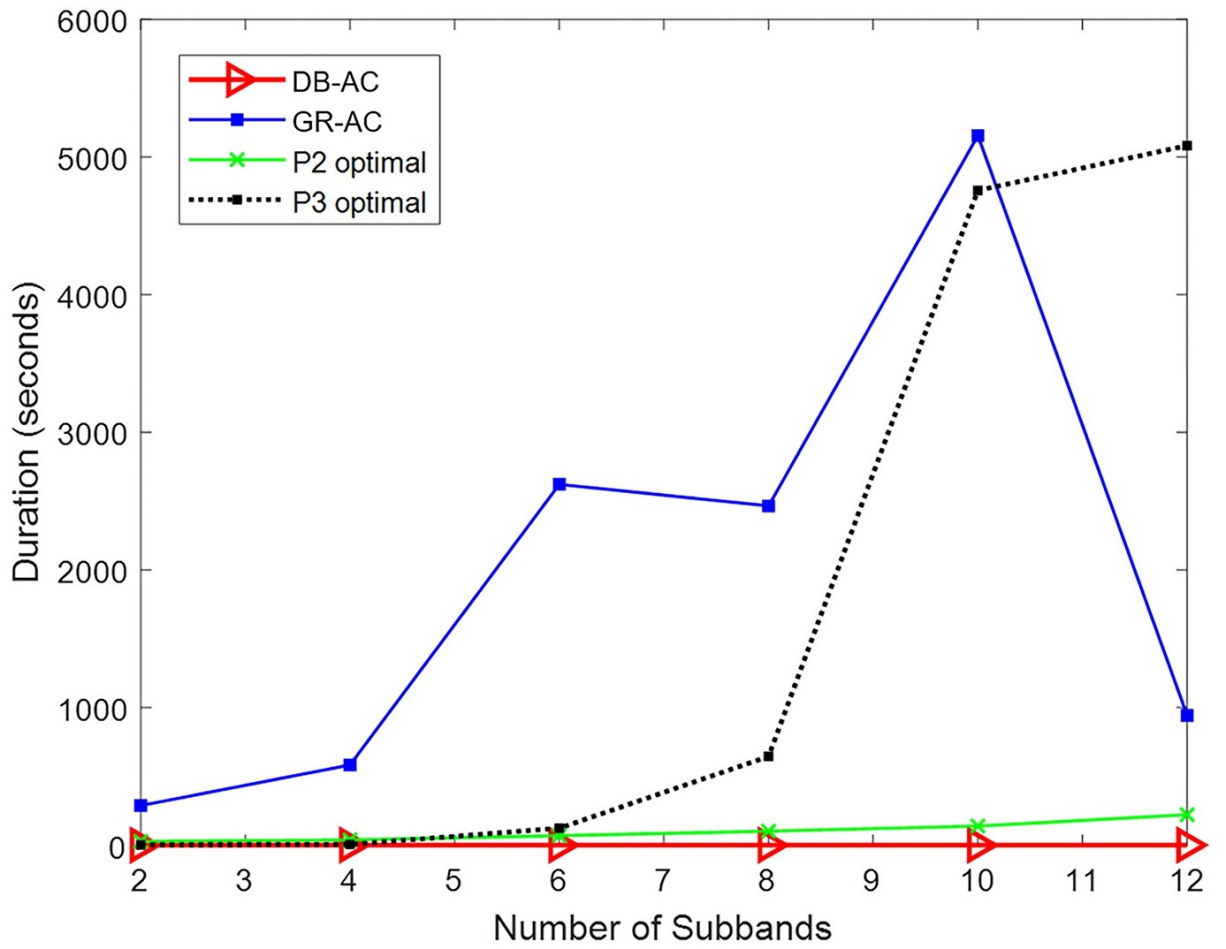
Time duration for obtaining answers to the four methods in different numbers of subbands.

The runtimes of the four methods are shown in Fig 4. The duration of the DB-AC algorithm, which is less than one second, is negligible compared to that of the other methods and does not change much as the size of the problem increases. The solution of P3 grows almost exponentially in time as the size of the problem increases. The GR-AC algorithm has a long runtime, but its growth pattern does not follow a specific behavior due to its heuristic nature. The solving time of P2, which is a convex problem, exhibits linear growth with a gentle slope as the number of subbands increases. According to Figs 3 and 4, it is evident that the proposed algorithm DB-AC performs well, reaching a nearly optimal solution in a relative short amount of time.

### 5.3. Examining the algorithm parameters

#### 5.3.1. Desirability function

In this section, the effect of the desirability function in the CNR algorithm on the result of the DB-AC algorithm is investigated. The desirability function determines how desirable a connection is from the BS’s viewpoint, and it is used when BS is deciding whether to keep or remove a connection among interfering connections.

**Fig 5:**
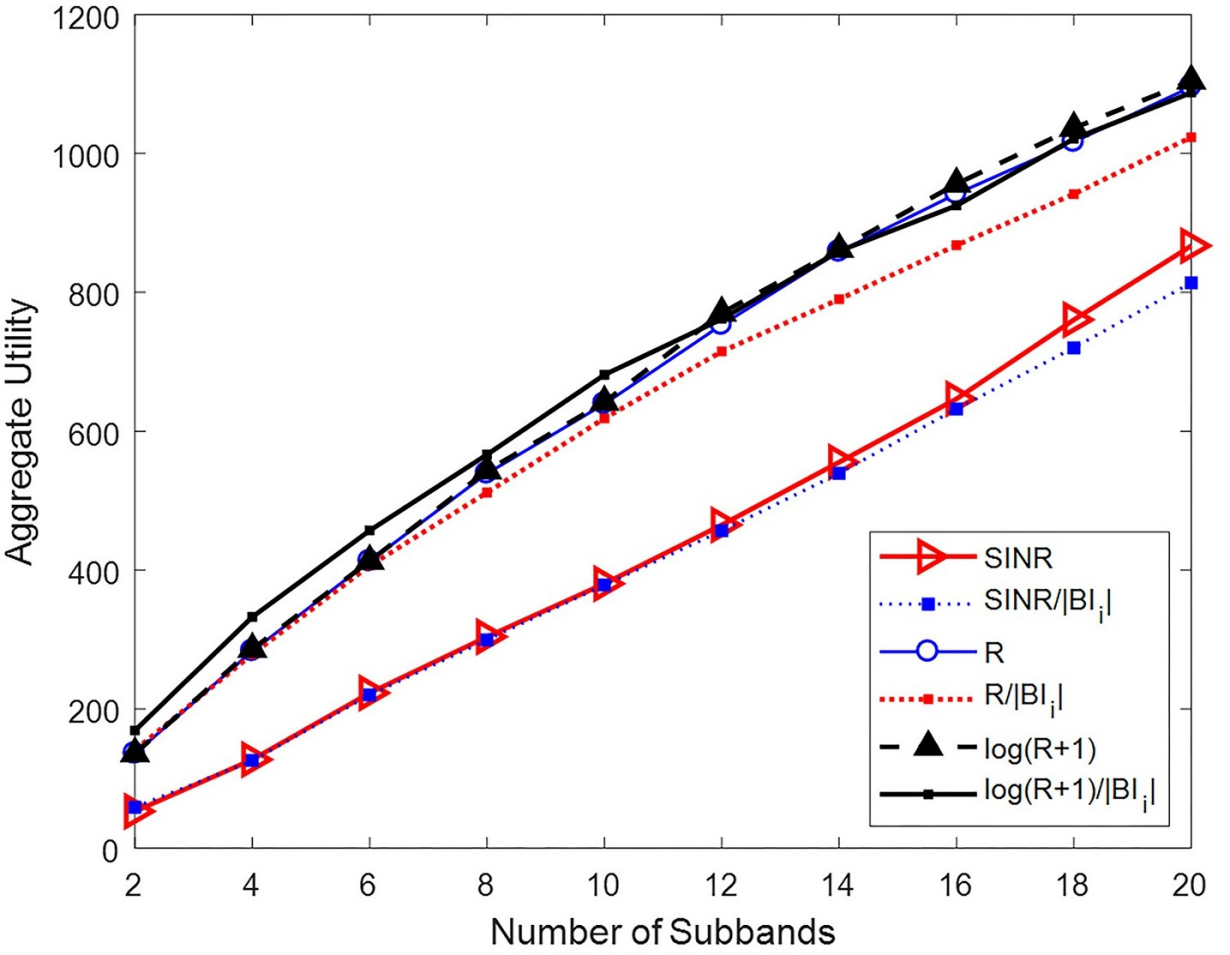
Network utility for different desirability functions.

A scenario with 200 users in HetNets with (1,3,6) BSs is considered. The number of network subbands is variable, and the network utility is obtained once the algorithm converges. The corresponding diagram is shown in Fig 5. In this diagram, each point is an average of 10 algorithm executions.

Based on Fig 5, in this scenario, the two desirability functions, $log(R_{ji}^{m}+1)$ and $log(R_{ji}^{m}+1)/\left|{BI}_{i}\right|$, yield higher network utility values compared with the desirability functions.

There is a slight difference in the algorithm results for these two desirability functions. Also, as shown in Fig 5, for a smaller number of subbands, the function $log(R_{ji}^{m}+1)/\left|{BI}_{i}\right|$ leads to a better result because it takes into account the size of each user interference set. Based on this criterion, users with a larger interference set are less desirable because establishing their connections will limit a relatively large number of network connections. However, by increasing the number of available subbands—and, therefore, increasing the network capacity—the desirability criterion $log(R_{ji}^{m}+1)$ leads to a better response. In the rest of the simulations presented in this research, ${des}_{ji}^{m}=\text{log}\left(R_{ji}^{m}+1\right)$ is considered.

#### 5.3.2. User selection list size

The size of the user selection list in the DB-AC algorithm can be set within the range of one to *B* × *M*. Due to the different number of BSs in each user interference area, the maximum size of a user *i* selection list equals $\left|{BI}_{i}\right|\times M$.

**Fig 6:**
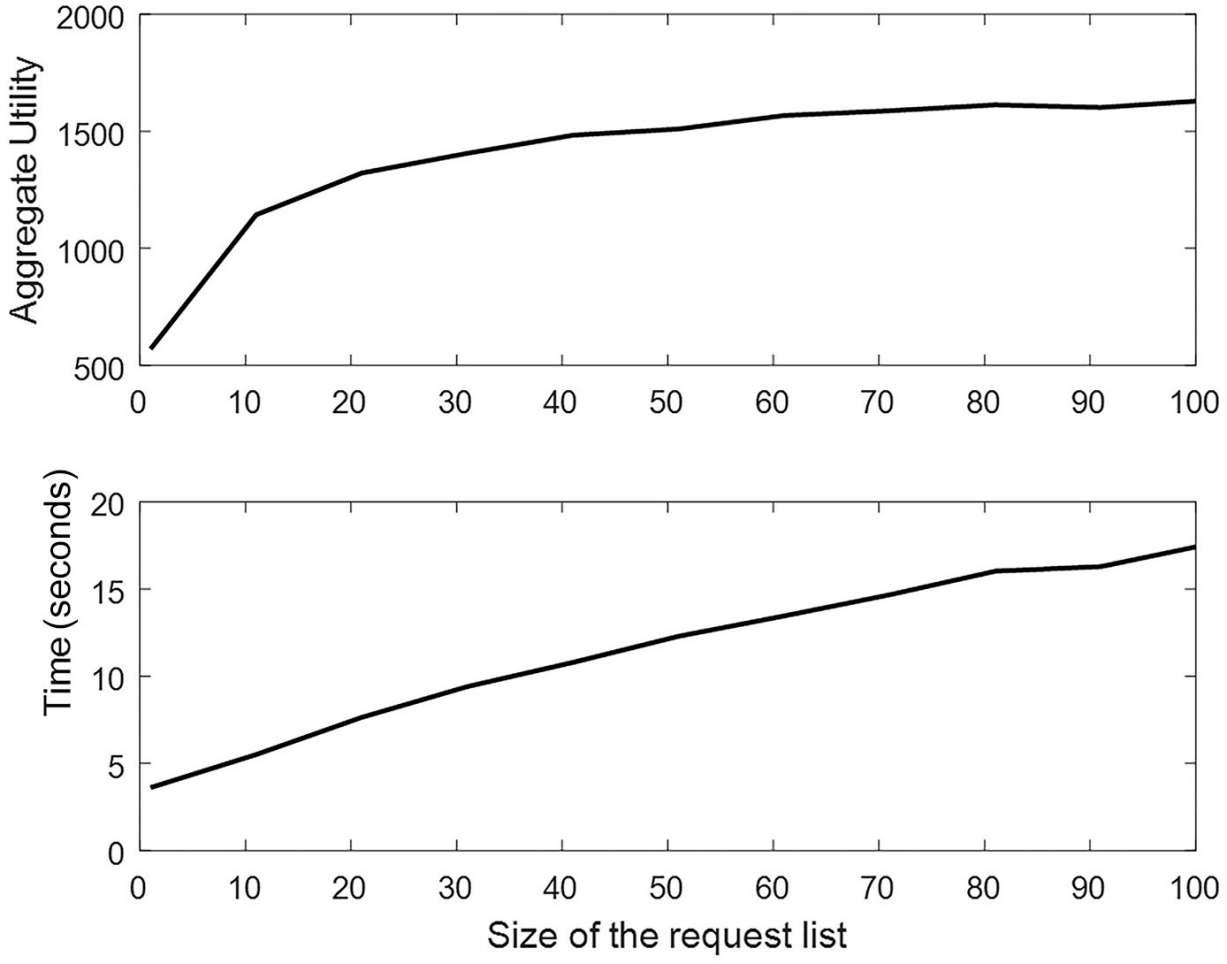
Network utility and algorithm runtime for different selection list size.

A network with (1, 6, 12) BSs, 200 users, and 20 subbands is considered to investigate the effect of the selection list size on the result of the algorithm and its runtime duration. The size of the selection list is expanded from 1 to 100, and the network utility and algorithm execution duration are measured. The corresponding diagram is shown in Fig 6. In this diagram, each point averages ten algorithm executions.

As shown in Fig 6, the total network utility grows as the selection list size increases. However, the execution time of the algorithm also increases as more examinations of user selections are required in the CNR heuristic algorithm. Due to the diminishing increase in network utility and the linear increase in runtime, it is necessary to select an appropriate size of the selection list for each network according to the trade-off between algorithm runtime and network utility. In this study’s simulation setups, a number between 20 and 50 was determined based on the target factor.

#### 5.3.3. Backhaul capacity

**Fig 7:**
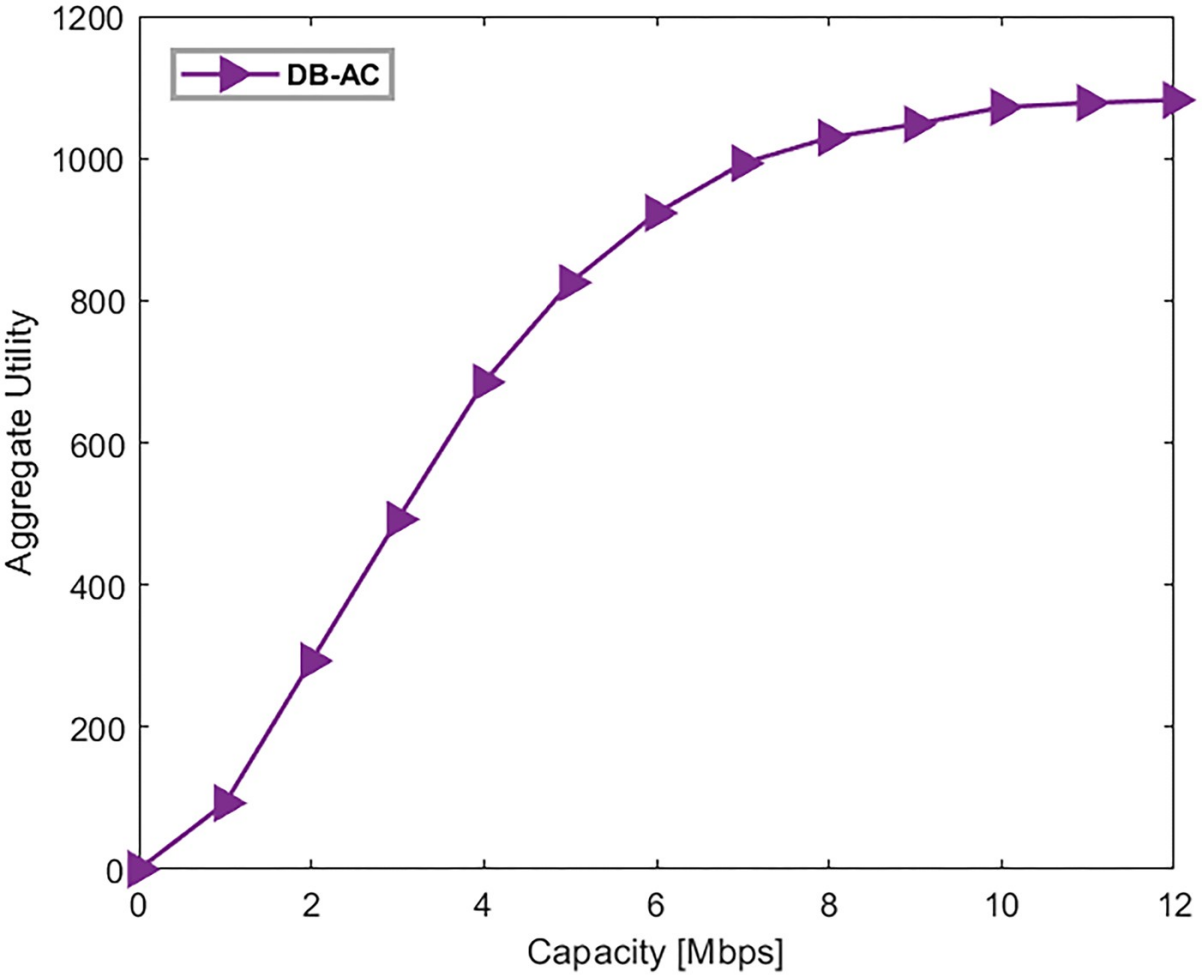
Network utility by changing the backhaul capacity of the BSs.

In this section, 100 users and (1,4,12) BSs, each providing 20 frequency subbands, are distributed in the network. In Fig 7, the backhaul connection capacity of the BSs is increasing. In this diagram, each point averages ten executions of the algorithm. Each point on the horizontal axis corresponds to the backhaul capacity of a femto BS, while pico and macro BSs have capacities 4 and 10 times greater, respectively. The total network utility grows by increasing the backhaul capacity of the BSs. However, the growth rate is decreasing and remains almost constant at 10 Mbps due to the limitation of the access network capacity. This highlights the importance of both the network’s backhaul link capacity and wireless access capacity.

**Fig 8:**
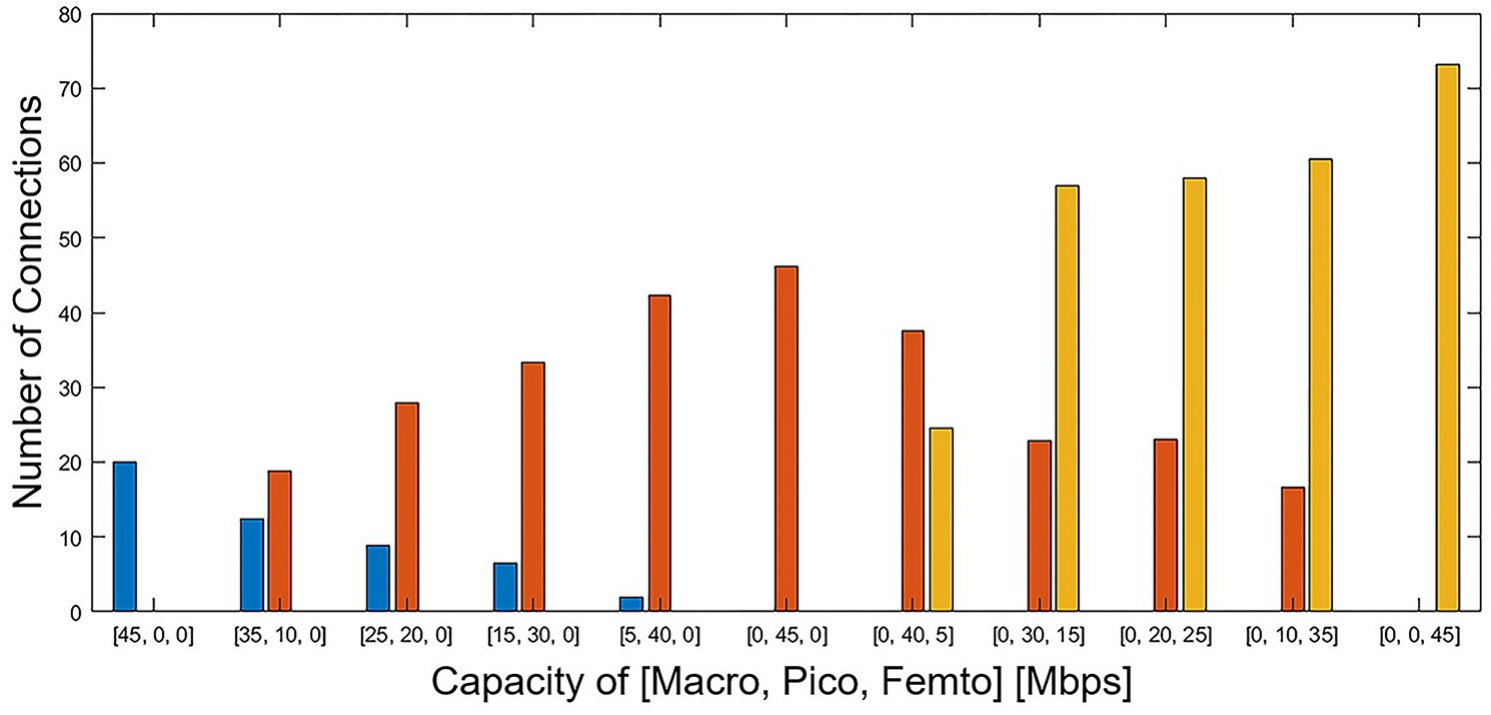
The number of connections by changing the backhaul capacity of BSs.

In the next scenario, the backhaul capacity of the BSs varies in a different manner. Each value on the horizontal axis shows the backhaul capacity of the macro, pico, and femto BS, respectively. As shown in Fig 8, limiting the backhaul connection capacity of BSs greatly affects the number of connected users. In the case where a high capacity is given only to the femto BSs, it leads to a large number of users connecting to them, given their higher quantity and dispersion.

Fig 8 illustrates how the number of connected users is influenced by both backhaul capacity and BS power. Initially, by increasing the capacity of the backhaul link, each type of BS has formed more connections. Considering the same backhaul link capacity, the number of connected users of each BS varies proportionally to the cell size (BS power), resulting in an average of 20, 11, and 5 connected users for each macro, pico, and femto BS, respectively.

#### 5.3.4. Users’ distribution

In this section, we examine the behavior of the algorithm under non-uniform user distributions, unlike other simulations with uniform random distributions. The users are distributed based on a gradient pattern, where the density of users varies gradually across the area. The parameter *α* controls the gradient pattern. Values greater than one for *α* cause a higher density of users near the macro BS and a gradually decreasing density as users move away from it. With the increase of *α*, the slope changes faster. On the other hand, values smaller than one for *α* cause a high density far from the center, around the space. In this case, the closer the *α* value is to zero, the steeper the slope of the changes. With *α* = 1, uniform distribution is achieved.

**Fig 9:**
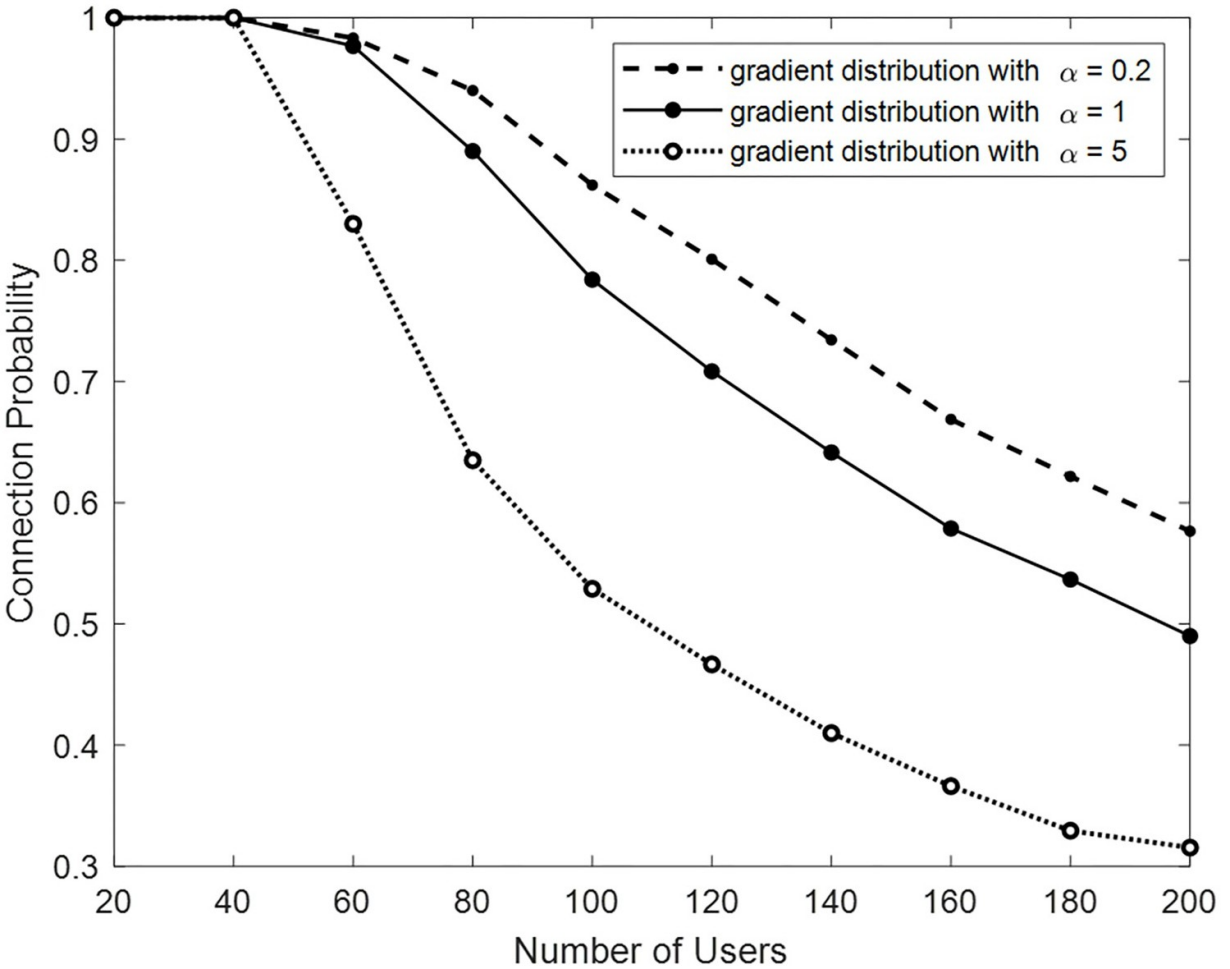
Connection probability by changing the number of users in different distributions.

In this scenario, there are (1,3,6) BSs, each providing 40 frequency subbands and each point is an average of 10 algorithm executions. Connections with intensive interference to each other are prevented by the interference protocol model and constraint in the system. Fig 9 illustrates that as the number of users and thus network load increase, the likelihood of user connection decreases, resulting from more rejections in the CNR algorithm.

In the case of gradient distribution with *α* = 5, due to the higher density of users in the center of space and around the macro BS and being far from the range of most small BSs, the capacity of small BSs has not been used well and the number of connections is less than that of other distributions.

In the case with *α* = 0.2, due to the distance of the users from the macro BS, the number of macro BS connections is lower than that in the uniform distribution mode (*α* = 1). The lower transmission power of small stations leads to a smaller interference range, enabling more frequency reuse and an increase in network connections. It’s important to note that the backhaul capacity limit of the BSs has not been applied in this scenario.

### 5.4. Performance comparison with the baseline methods

#### 5.4.1. In the case of no backhaul capacity limitation

In this section, interference, load balancing among tiers, and network utility are investigated. The simulation results of the DB-AC algorithm reveal that the total harmful interference for each active connection amount to zero. In other words, there is no harmful interference, either within or between tiers, for active connections. This outcome is a result of the interference constraint in the P3 optimization problem and the use of the CNR algorithm in the distributed algorithm.

In this paper, as in [19], assuming that all users always have data for reception, the load of each cell is considered equal to the number of users of that cell. We examine the distribution of users among cells of different tiers, by analyzing the number of users assigned to the BSs of each tier for our distributed algorithm and two common cell association methods. In this scenario, a network with (1,3,6) BSs, 100 users, and 20 subbands is considered.

**Fig 10:**
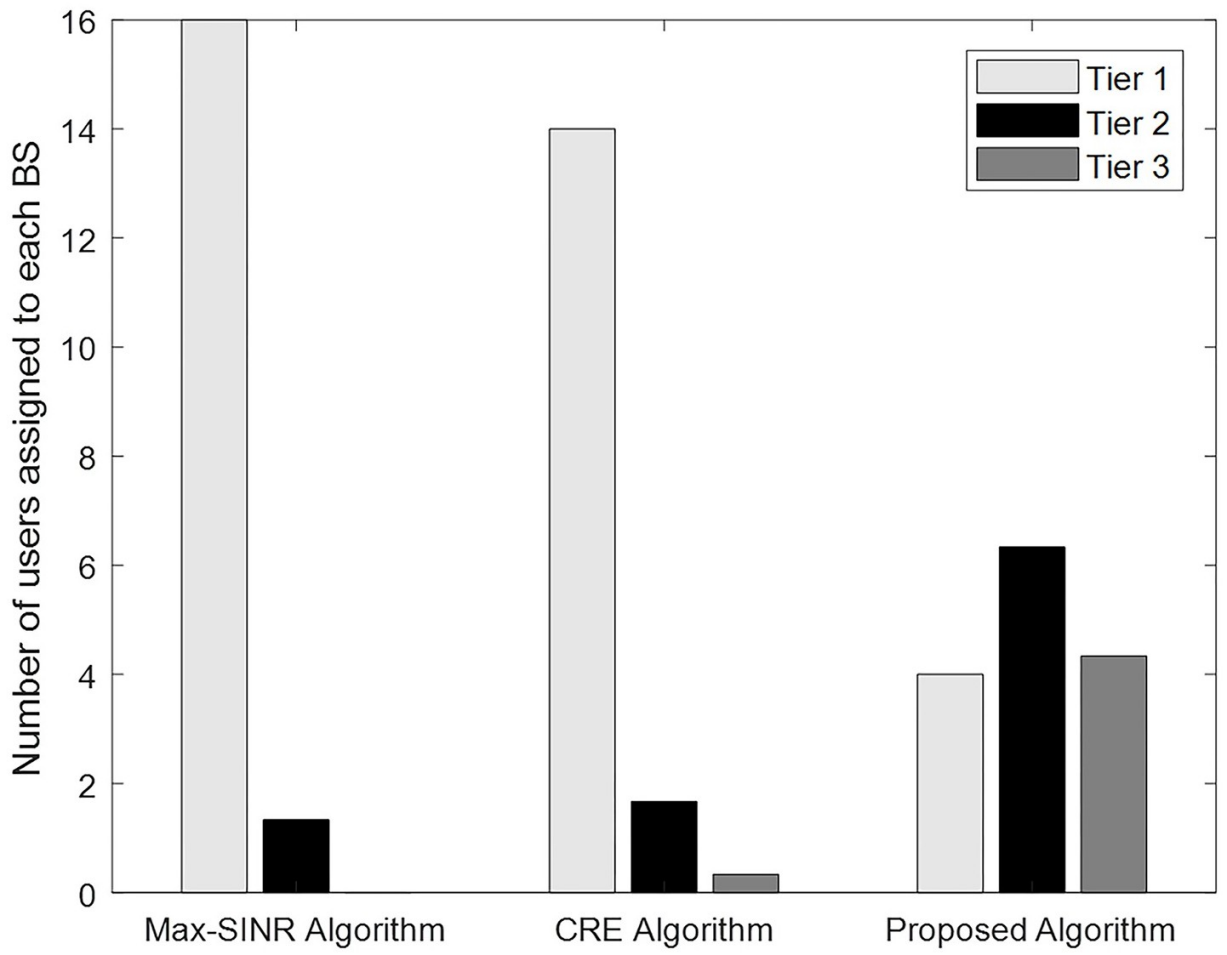
Average number of user connections for each BS in each tier.

For the evaluation of our algorithm under different network scenarios, max-SINR and cell range expansion (CRE) methods were chosen. These methods are both widely recognized cell association schemes and have been referred in recent research [71]. In the max-SINR, each user is associated with the BS with the maximum received SINR. In the CRE approach, which is one of the common load balancing methods in HetNets, the user first biases the received SINR from BSs. The BS with the maximum biased SINR is selected for connection [13, 39]. Fig 10 compares the numbers of users served by each BS in each tier for different cell association methods.

As shown in Fig 10, in the max-SINR allocation method, many users are assigned to the macro cell due to the higher transmission power of the macro BS, causing a severe load imbalance in the network. In this case, most small-scale BSs will have no network connection. The CRE method has led more users to smaller cells by biasing the received SINR with bias coefficients (1, 4, and 6 for macro, pico, and femto BSs, respectively).

It can be observed from Fig 10, our proposed scheme results in more users being connected to smaller BSs than in the previous methods, improving load balance across network tiers. This reduces the load imposed on the macro cell and pushes users onto lightly loaded small-scale BSs to improve total network utility.

It should also be noted that the proposed distributed algorithm has a longer runtime than conventional SINR-based methods. This feature makes this algorithm suitable for allocating cells and subbands in networks with low-mobility environments.

**Fig 11:**
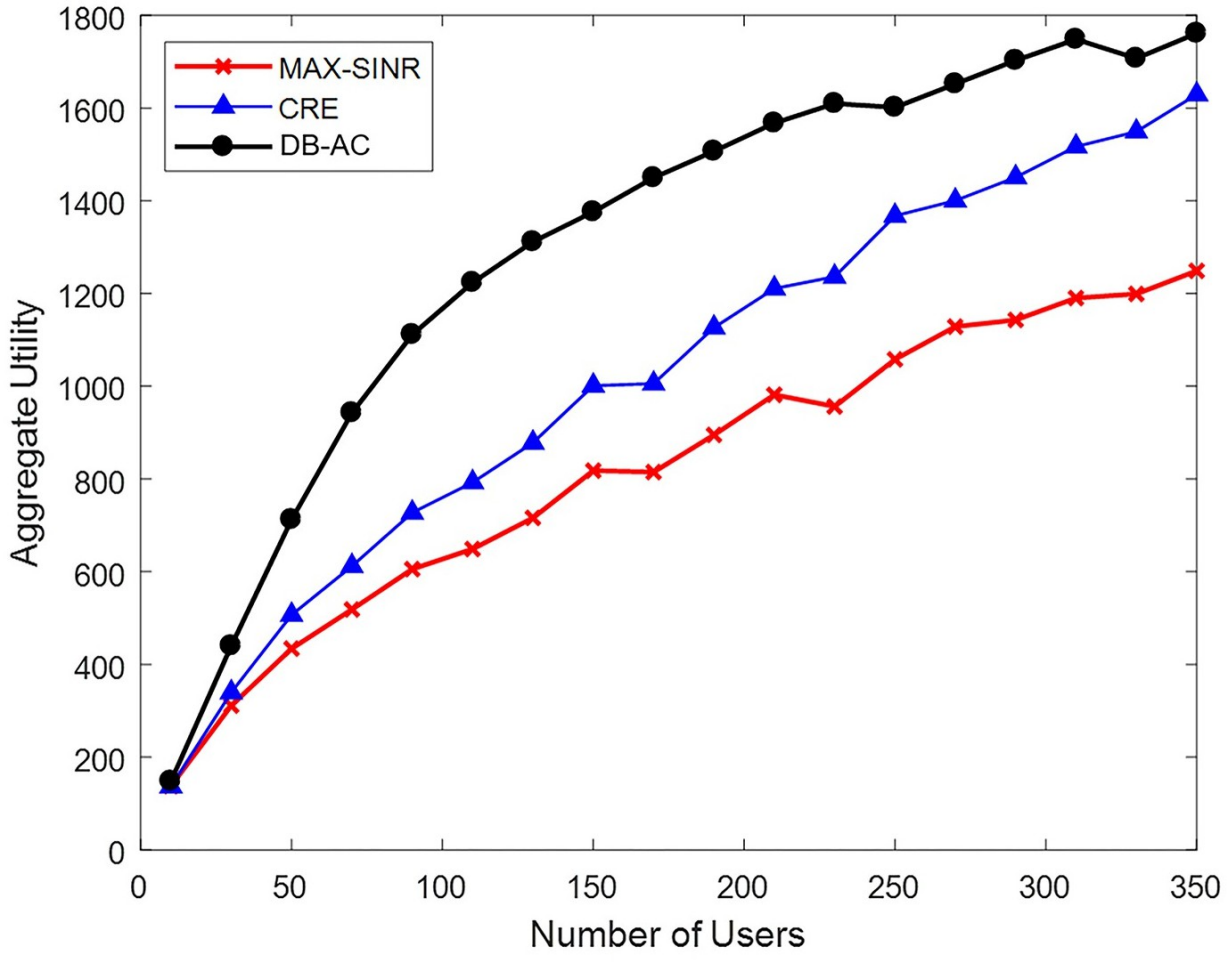
Network utility by changing the number of users.

A scenario with (1,6,12) BSs, each with 20 subbands, is developed to investigate the effects of an increasing number of network users. The size of the user selection list is 30. Diagrams of the network utility of the proposed algorithm (DB-AC), max-SINR, and CRE methods are illustrated in Fig 11. Each point is an average of ten algorithm executions. The total network utility of the proposed algorithm is significantly higher than that of the max-SINR and CRE methods.

Fig 11 also shows that as the number of users in the network increases, the total network utility of the proposed algorithm increases. However, the rate of utility growth gradually diminishes due to limited network capacity.

**Fig 12:**
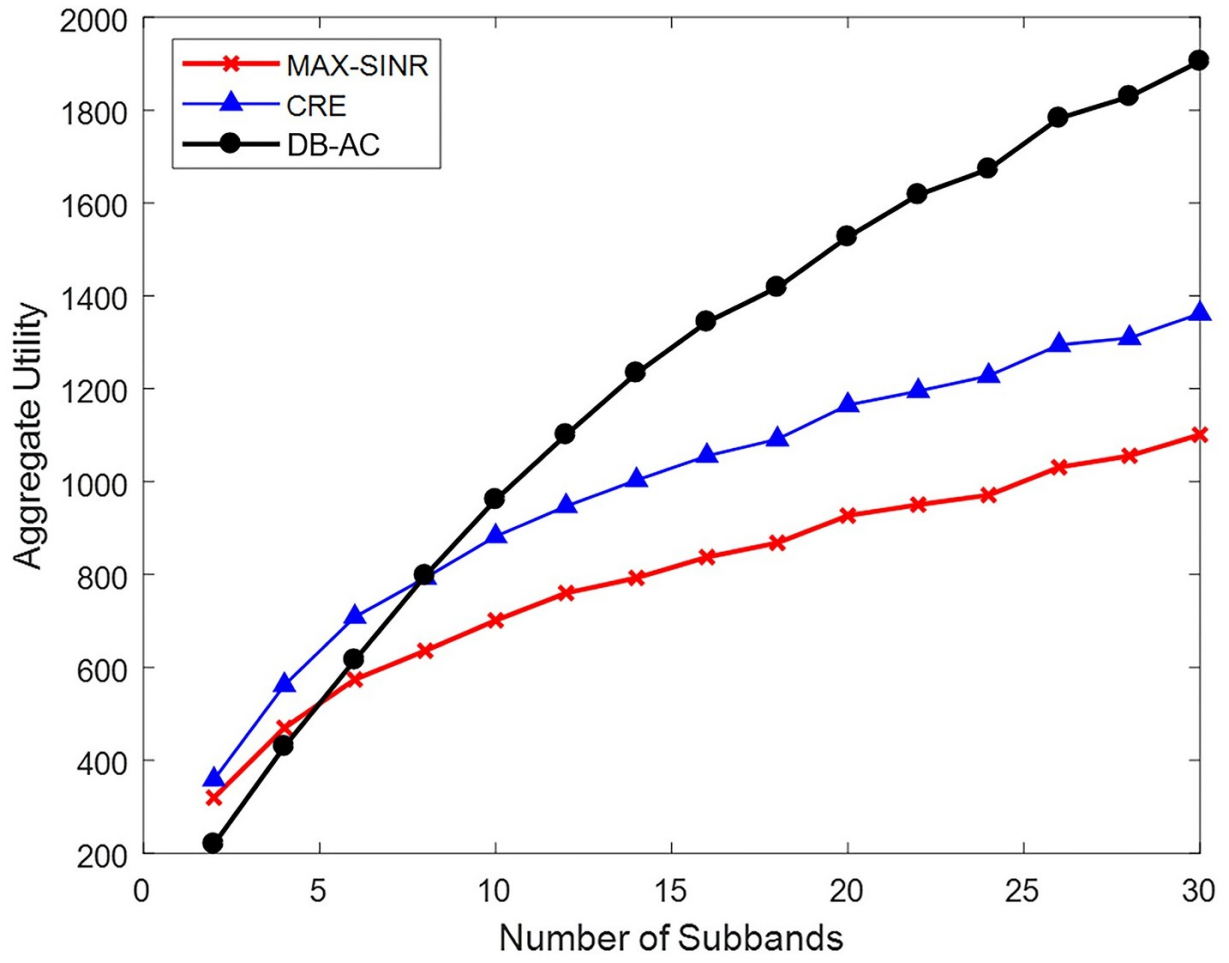
Network utility by changing the number of subbands of each BS.

In this scenario, the effect of increasing the number of subbands and therefore network resources on the behavior of the proposed algorithm, max-SINR, and CRE methods is explored (see Fig 12). In this scenario, 200 users are placed in a network with (1,6,12) BSs. The size of the user selection list is 30. In this diagram, each point is an average of 10 algorithm executions.

As shown in Fig 12, as the number of subbands of each BS increases, more users in the network can be serviced. Therefore, the total network utility increases for all three methods. The max-SINR and CRE methods excel with few subbands because due to the lack of interference constraints, they enable the use of the same subband for multiple interfering connections. However, as the number of subbands increases, the network utility of the DB-AC algorithm is significantly higher than that of the max-SINR and CRE methods. This difference increases further as the number of subbands increases.

The max-SINR and CRE methods are both one-step schemes. Despite their low execution time, they do not necessarily establish proper connections. In addition, with an increase in the number of subbands, they cannot make optimal use of the network’s added capacity. The proposed algorithm is an iterative algorithm that improves the connections in each iteration and better utilizes the remaining network capacity.

#### 5.4.2. In the case of backhaul capacity limitation

For further investigation, two relevant cell allocation methods, "Q-learning"[40] and "NSGA-II" [72], have also been investigated. A scenario with one macro BS and 20 small-scale BSs is established. The backhaul capacity for macro BS is 50 Mbps and for smaller BSs is a random number in the range [1.5,10] Mbps. The power status and channel conditions are similar to [40].

**Fig 13:**
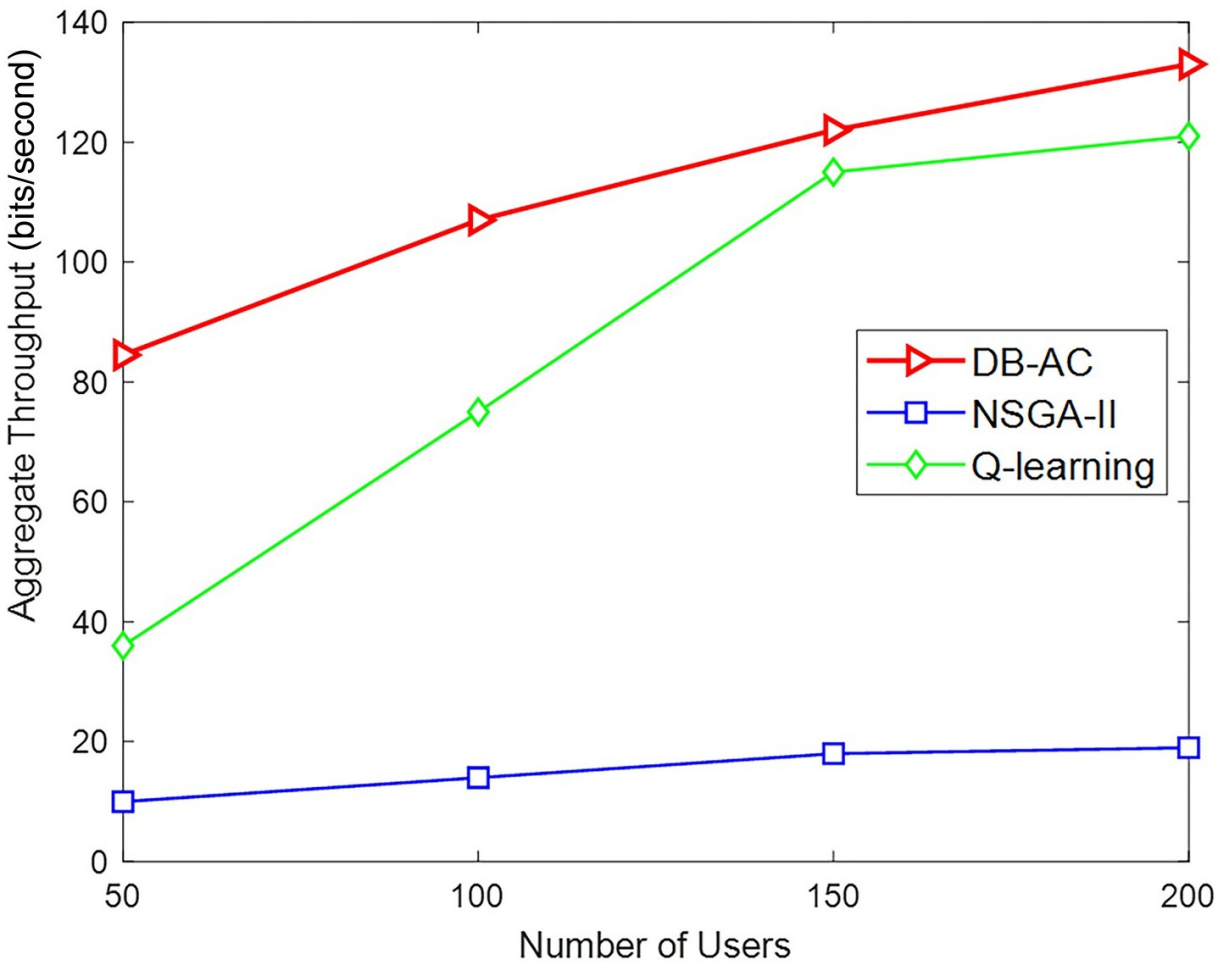
Aggregate throughput by changing the number of users.

**Fig 14:**
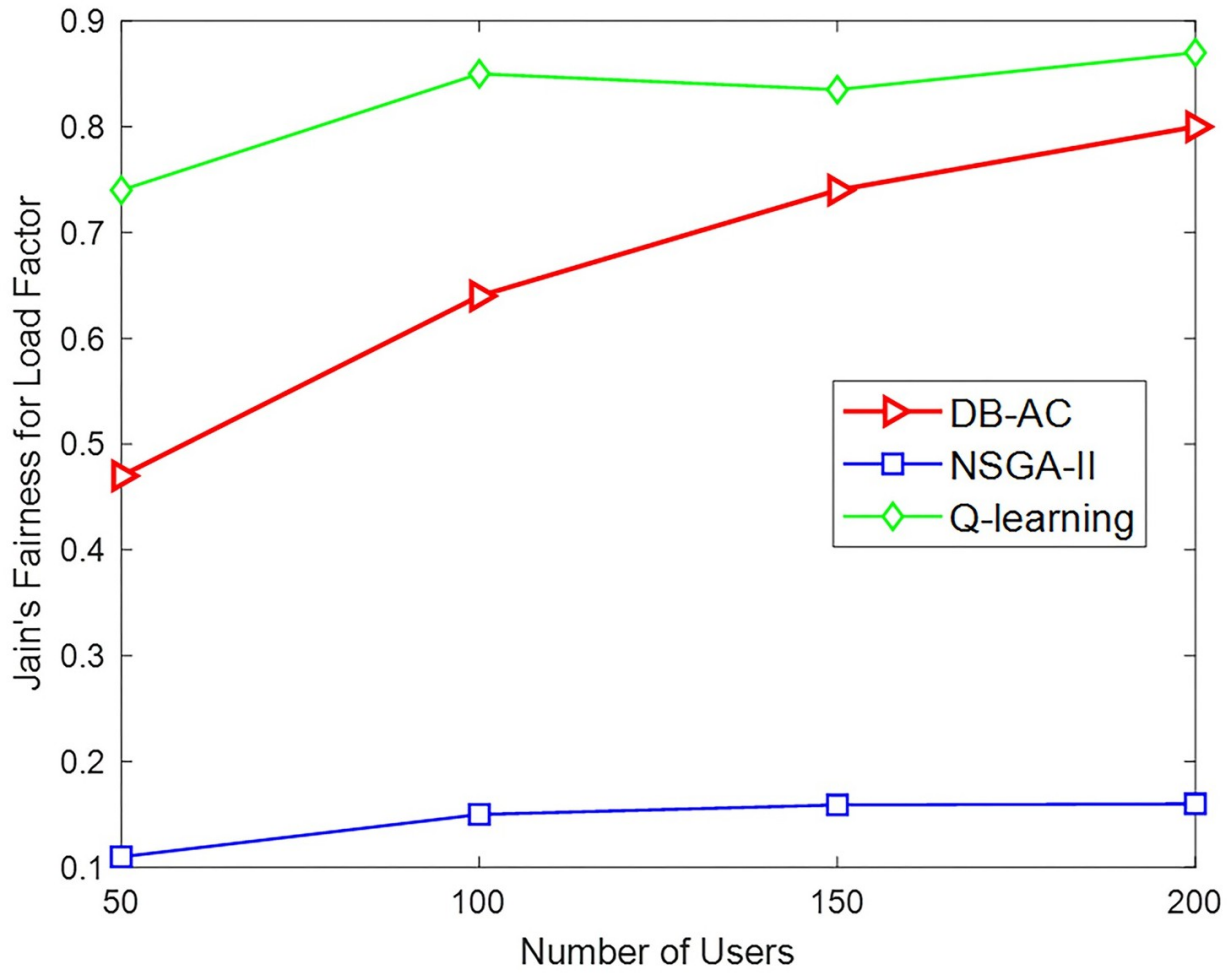
Jain’s fairness for load factor by changing the number of users.

For each BS *j*, a relative load factor *l*_*j*_ is defined, which is the total data rate for its connected users compared to the capacity of its backhaul link. Here, Jain’s fairness index, defined as $\frac{{\left(\sum_{j\in B}l_{j}\right)}^{2}}{B\sum_{j\in B}{l_{j}}^{2}}$ is adopted. Figs 13 and 14 compare the proposed algorithm with the two related methods. The DB-AC algorithm, along with being distributed, has been able to increase the total throughput of network users. In fact, our proposed method not only increases the network utility, but also improves the total network throughput. As shown from Fig 14, the load factor distribution of the BSs in DB-AC is much higher than in “NSGA-II” but less in compared to “Q-learning” method, which happened due to the lack of connection for some small BSs in some executions.

## 6. Conclusion

This paper discusses a practical solution for user association in backhaul-constrained HetNets. The methods proposed in this study, along with user association, aim to achieve load balancing and inter-cell interference management. As we discussed, meeting these goals is necessary for achieving utility gain and throughput enhancement in HetNets. In this study, instead of relying on protected macro cell resource blocks, which only manages a specific group of inter-cell interference, managing interference between all BSs is considered. Thus, the protocol interference model and interference constraints are used in system modeling and problem formulation. The solutions proposed in this study protect network connections against any intense interference.

According to the simulation results, the GR-AC algorithm offers a suboptimal solution to the cell and subband allocation problem. Meanwhile, the distributed DB-AC algorithm delivers a nearly optimal response at the appropriate time. Despite the higher message exchange rate and runtime duration of the proposed distributed algorithm compared to conventional SINR-based methods, simulation results demonstrate a significant improvement in network utility, except for scenarios with few subbands. In general, our distributed solution effectively utilizes the increased capacity brought by small-scale BSs in heterogeneous networks.

The DB-AC algorithm relies on local computations and local information only. This condition, along with the efficiency of the algorithm, makes it appropriate for implementation in practical environments. This study considers a general system model with no limitation on the number of tiers, BSs, or indoor/outdoor conditions, and also allows for differences in capacity limits of the backhaul networks of the BSs. Therefore, the proposed solution can be employed in different HetNets scenarios.
